# Markers of the ageing macrophage: a systematic review and meta-analysis

**DOI:** 10.3389/fimmu.2023.1222308

**Published:** 2023-07-13

**Authors:** Charlotte E. Moss, Hew Phipps, Heather L. Wilson, Endre Kiss-Toth

**Affiliations:** ^1^ Department of Infection, Immunity and Cardiovascular Disease, Medical School, University of Sheffield, Sheffield, United Kingdom; ^2^ Healthy Lifespan Institute, University of Sheffield, Sheffield, United Kingdom

**Keywords:** macrophage, ageing, meta-analysis, infammageing, immunity

## Abstract

**Introduction:**

Ageing research is establishing macrophages as key immune system regulators that undergo functional decline. Due to heterogeneity between species and tissue populations, a plethora of data exist and the power of scientific conclusions can vary substantially. This meta-analysis by information content (MAIC) and systematic literature review (SLR) aims to determine overall changes in macrophage gene and protein expression, as well as function, with age.

**Methods:**

PubMed was utilized to collate peer-reviewed literature relating to macrophage ageing. Primary studies comparing macrophages in at least two age groups were included. Data pertaining to gene or protein expression alongside method used were extracted for MAIC analysis. For SLR analysis, data included all macrophage-specific changes with age, as well as species, ontogeny and age of groups assessed.

**Results:**

A total of 240 studies were included; 122 of which qualified for MAIC. The majority of papers focussed on changes in macrophage count/infiltration as a function of age, followed by gene and protein expression. The MAIC found iNOS and TNF to be the most commonly investigated entities, with 328 genes and 175 proteins showing consistent dysregulation with age across the literature. Overall findings indicate that cytokine secretion and phagocytosis are reduced and reactive oxygen species production is increased in the ageing macrophage.

**Discussion:**

Collectively, our analysis identifies critical regulators in macrophage ageing that are consistently dysregulated, highlighting a plethora of targets for further investigation. Consistent functional changes with age found here can be used to confirm an ageing macrophage phenotype in specific studies and experimental models.

## 1 Introduction

Largely due to improvements in healthcare and living standards, those in the developed world are living longer, with people aged over 65 years making up nearly 20% of the UK population in 2021, compared with less than 11% in 1950 (1, 2). However, this increase in lifespan does not correlate with healthspan, thus much of our elderly population are living with poor quality of life, increasing pressure on societal resources including health and social care. Unhealthy ageing is often described in terms of multimorbidity, the presence of more than one age-related disease (ARD), with each additional ARD increasing the risk of developing another (3). There is a wide overlap in the causes of developing ARDs, as they occur in no particular order and eventually affect many different organ systems (4). Thus, geroscience research is steadily gaining ground, with the aim of finding biomarkers and therapeutic interventions to prevent progression of multimorbidity and the associated decrease in quality of life (5).

More recent advances have noted the crucial role of immune system functional decline in the progression of ARDs, in both innate and adaptive immune cells (6). This is often referred to as immunosenescence (7) and has been linked to a reduced immune response to new and previously encountered infections, as well as vaccines (8). The functional decline of the adaptive immune system, made up primarily of B-, T- and dendritic cells, is already well characterised (9–13). The innate immune system is the first responder to pathogens, often starting with recognition by neutrophils and macrophages that go on to initiate the adaptive immune response (14). Neutrophil phagocytosis and reactive oxygen species (ROS) production has been found to decline with age (15). Neutrophil extracellular traps have also been shown to be defective with increasing age, both reducing their bactericidal activity (16). Macrophages also undergo functional decline with age, and this will be discussed in depth throughout this review.

Macrophages are a diverse set of innate immune cells, collectively known for their phagocytic ability (17). They are uniquely plastic in nature, making them able to shift and reprogram in response to environmental cues (18, 19). This puts them at the forefront of mounting an immune response, enabling them to further inform their surroundings through cytokine and chemokine production (20). Macrophages also largely govern inflammation, at both the initiation and resolution phase (21). These macrophage characteristics are largely dependent on stimuli within their microenvironment and different tissue macrophage populations can have widely different functions and appear phenotypically distinct at the transcriptional level (22). Although the role of macrophages within different tissues is now largely understood, knowledge is lacking in the context of ageing, and there is a pressing need for research data to be collated and consolidated. Our current knowledge of macrophages in ageing tissues centres around inflammaging, a term used to describe the development of a persistent low-grade, sterile inflammatory phenotype that develops with age (23). One possible cause for this is macrophage chronic activation, leading to production of more inflammatory cytokines that affect neighbouring cells and the tissue environment (24). Chronic activation of the immune system is energetically costly, and the resultant effect is an inability to mount a full immune response and therefore fight off pathogens, much like that of immunosenescence (7, 25).

As macrophages have now been implicated in age-related immune system decline and inflammaging, an increasing body of data assessing macrophage changes with age are being reported. However, data largely remain unstandardised, where the cellular ontogeny and tissue location proving crucial to corresponding function, reaction to stimuli, surface marker expression or cytokine secretion (22, 26). This review therefore aims to consolidate current knowledge on the ageing macrophage, in a way that allows for comparison between macrophage populations from different mammalian species and tissues. Specifically, it aims to highlight where there is consistency in changes in gene and protein expression during ageing and our current knowledge of tissue- and species-specific functional changes in the macrophage with age. We will present and discuss the role of macrophages in ageing through a narrative review with a systematic approach as well as findings from a meta-analysis by information content (MAIC) in order to provide a comprehensive list of genes and proteins dysregulated with age, a useful tool both for modelling macrophage ageing and potential therapeutic targets for age-related functional decline.

## 2 Methods

The SLR and MAIC presented here were conducted adhering to PRISMA 2020 guidelines (27).

### 2.1 Search strategy

Two independent reviewers searched PubMed up to March 2022 for MAIC and December 2022 for SLR using the following search term:

**Table:** 

((((Macrophage[Title/Abstract]) AND ((Ageing[Title/Abstract]) OR (Aging[Title/Abstract]))) OR ((Macrophage[Title/Abstract]) AND (Old[Title/Abstract])) OR ((Macrophage[Title/Abstract]) AND (Longevity[Title/Abstract])) OR ((Macrophage[Title/Abstract]) AND (Age[Title/Abstract])))

Any articles not in English and any duplicates were then removed.

### 2.2 Eligibility and selection process

In this review, studies were included if they met the following criteria:

1. Full-text article available in English
2. Primary research article
3. At least one set of results directly comparing young vs. old samples, with old being defined by the article authors
4. At least one set of results directly assessing isolated macrophages
  Additionally, studies were further marked for MAIC if they met the final criteria:
5. Data directly assessing a named gene or protein’s expression in young and old macrophages

Where reviewers had differing opinions, studies were discussed in more depth until a consensus decision could be made. Both reviewers’ selections were then combined.

### 2.3 Data extraction for systematic literature review

Data extraction for the SLR included the following information:

1. Whether the article fit the SLR, MAIC criteria, or both
2. General article information, such as the title, authors and study design
3. Experimental design including species looked at, type of macrophages assessed, age of groups assessed and experimental techniques used
4. Results pertaining to macrophage ageing

### 2.4 Data extraction for meta-analysis by information content

**Figure 1:**
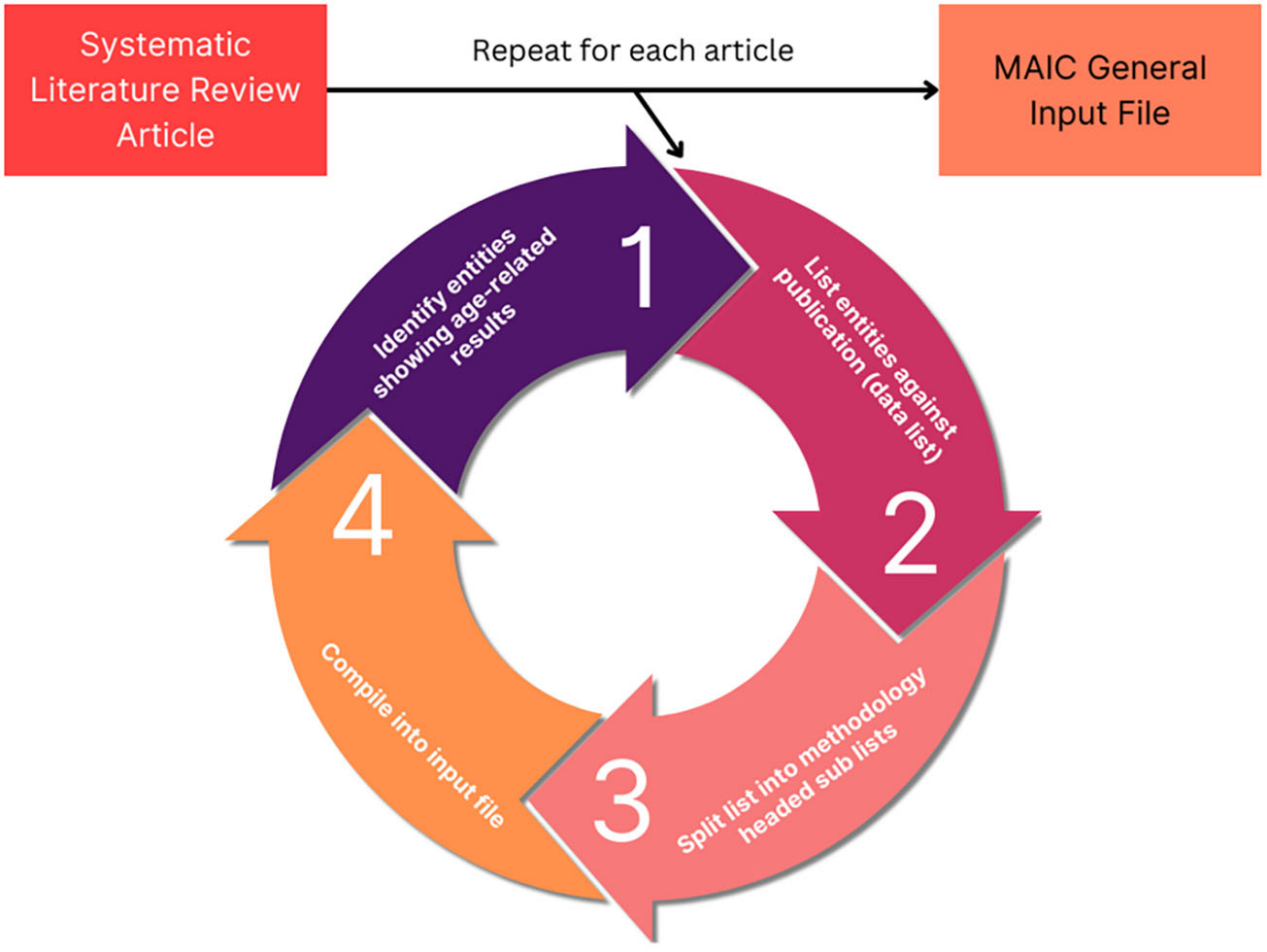
MAIC input data processing. The analysis process used to obtain MAIC data. The cycle is repeated for each qualifying study, meeting the inclusion criteria. Each list of data is placed into a tab delimited.txt file stacking into a super list with each data list occupying a row. In the case of the other 4 input files (genes upregulated, genes downregulated, proteins upregulated, proteins downregulated), “entity” is replaced with the respective data element, such as genes upregulated with age.

The MAIC algorithm used was developed by the Baillie Lab (28, 29). The online server and user interface is available at https://baillielab.net/maic. Qualifying articles listing gene/protein hits were compiled into a text file to create a database (**Figure 1**). This database was indexed by separating each publication into lists based on the method used to obtain the gene/protein data. If a publication presented experimental results from three methods, the result would be three lists of the respective genes/proteins titled [Xa], [Xb], [Xc] and headed by their respective method.

Separate input files were generated in order to differentiate between genes and proteins, as well as whether expression was up- or downregulated with age, resulting in five separate input files:

1. All Entities (Genes and Proteins)
2. Genes Upregulated
3. Genes Downregulated
4. Proteins Increased
5. Proteins Decreased

The MAIC algorithm was executed on each input file using Python and output data were processed in Rstudio.

## 3 Results

### 3.1 Stratifying the literature for MAIC and SLR analyses

**Figure 2:**
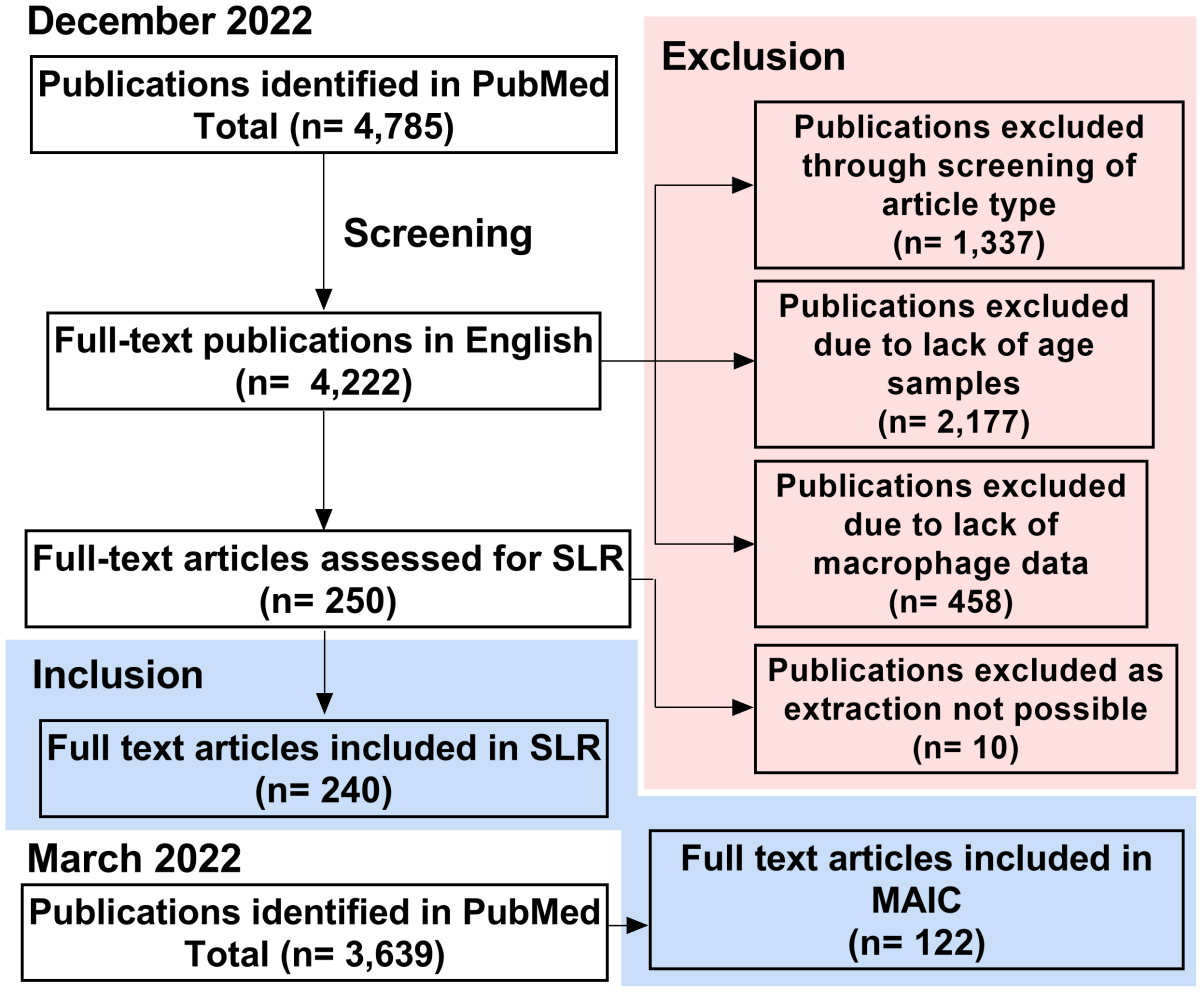
Study screening and selection process. Process of study inclusion and exclusion for SLR (completed December 2022) and MAIC (completed March 2022). SLR, systematic literature review; MAIC, meta-analysis by information content.

An initial search of PubMed identified 4,785 papers, relating to macrophages and ageing, as of December 2022 (**Figure 2**). This was refined to 4,222 full-text publications in English. 1,337 publications were excluded due to not containing primary research or being case reports. A further 2,177 publications were excluded due to lack of comparison between young and aged groups and 458 due to not containing macrophage-specific data on further inspection. This left 250 primary research articles that met all criteria for the systematic literature review. During data extraction, a further 10 papers were excluded due to lack of availability, meaning 240 publications were included in this SLR. PubMed search was completed in March 2022 for the MAIC, at which time 3,639 articles were identified by our search strategy. 122 of these articles met the more stringent inclusion criteria, containing data pertaining to changes in genes or protein expression in the macrophage with age.

### 3.2 Systematic literature review of the ageing macrophage

**Figure 3:**
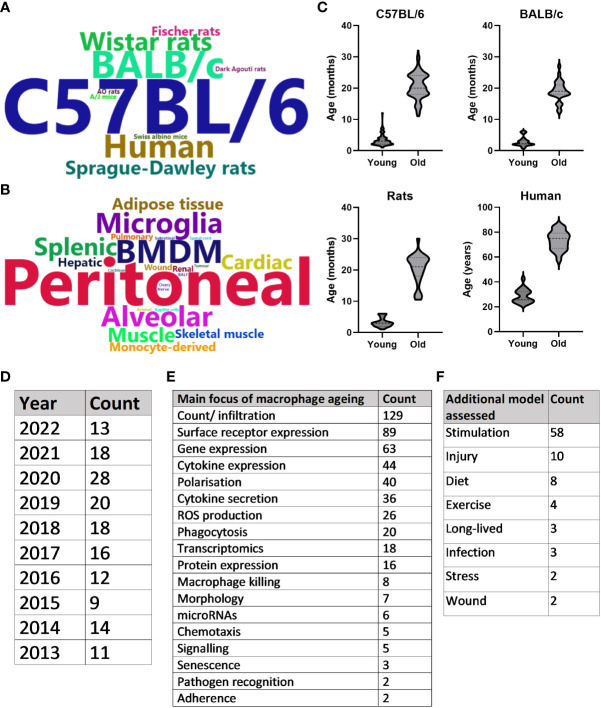
Systematic literature review summary. **(A)** Word cloud of most commonly assessed species in the context of macrophage ageing. Words are sized based on the frequency that the species is studied in the literature. **(B)** Word cloud of most commonly assessed macrophage types. Words are sized based on the frequency that the macrophage type is studied in the literature. **(C)** Violin plots of the ages assessed when comparing young and old macrophages of the four most common species studied in the literature. Either mean age stated in the publication or median of age range was used. **(D)** Number of included publications produced per year for the past 10 years. **(E)** Counts of the main focus for results of each included publication. **(F)** Counts of any disease model assessed in the publication in addition to healthy macrophage ageing.

A summary of the publications that met inclusion criteria for the SLR can be found in **Figure 3**. Overall, the search strategy identified 240 unique primary research articles for data extraction, as described above. The majority of publications used C57BL/6 mice to study ageing (n = 155), with BALB/c mice (n = 25), humans (n = 17) and Wistar rats (n = 12) being the next most frequently used species (**Figure 3A**). Publications were further characterised by macrophage population used, whereby peritoneal (n = 82), bone marrow-derived (n = 38), alveolar (n = 17) and microglia (n = 16) were the most common macrophage populations to be assessed (**Figure 3B**). **Figure 3C** highlights the ages most commonly assessed when comparing young and old of the most frequently assessed species. For this, the mean age was used when stated in the publication and when an age range was stated, the median was used. For each species, “young” was more clearly defined, as evidenced by the flatter violins: mean age for C57BL/6 mice was 3.4 months (95% CI: 3.1-3.7), for BALB/c mice was 2.9 months (95% CI: 2.3-3.4), for rats was 3.2 months (95% CI: 2.6-3.8) and for humans was 28.8 years (95% CI: 26.13-31.42). C57BL/6 aged mice were especially varied, with 2 peaks at approximately 18 and 24 months. The mean age here was 20.7 months (95% CI: 20-21.32), for BALB/c mice was 19 months (95% CI: 17.8-20.3), for rats was 20.4 months (95% CI: 18.4-22.5) and for humans was 73.3 years (69.7-76.9). The number of annual publications for the past 10 years that were included in this SLR is shown in **Figure 3D**. Although 2020 saw an increase in publications, this number has remained broadly consistent for the past 10 years. **Figures 3E, F** depicts the overall focus of the publications, with the most common focus of macrophage ageing being changes in overall numbers of resident or infiltrating cells, frequently examined by macrophage surface receptor expression. This was often analysed in conjunction with assessing how aged macrophages differ compared with young in their response to stimuli, such as lipopolysaccharide (LPS) or polarising cytokines. Data from these publications are discussed in more detail below.

#### 3.2.1 Macrophage infiltration in response to age

**Figure 4:**
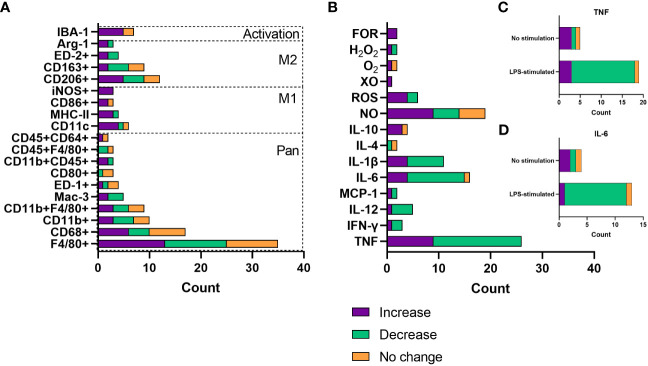
Changes with age in macrophage count/infiltration and soluble mediator release. **(A)** Number of publications assessing changes with age in macrophage infiltration or count by surface marker expression. **(B)** Number of publications assessing changes with age in soluble mediator release, including cytokines and reactive oxygen species. Data are only included where there were results from more than one publication. **(C)** Number of publications assessing TNF release as a consequence of age, grouped by whether cells were stimulated with LPS or not stimulated. **(D)** Number of publications assessing TNF release as a consequence of age, grouped by whether cells were stimulated with LPS or not stimulated. FOR, free oxygen radicals; XO, xanthine oxidase; ROS, reactive oxygen species; NO, nitric oxide.

Resident macrophage count and infiltration of primary macrophages were by far the most frequent focus when assessing changes with increasing age. For this, most studies analysed surface marker expression by flow cytometry or immunohistochemistry. There are a plethora of different macrophage markers with species- and tissue-specificity, as well as those that indicate the inflammatory phenotype of the cell (22, 30). Data were extracted from publications that assessed these age-related changes and collated by the surface markers assessed. Whether these macrophage populations increased, decreased or underwent no change with age was then collated (**Figure 4A**). The species, tissue and macrophage subtype assessed, as well as the publication that the data pertains to, are shown in **Supplementary Table 1**. According to the literature pool, most commonly tissue macrophage populations were assessed, with pan-macrophage markers making up the bulk of the surface marker expression data. There was no particular trend in whether these cells increased, decreased or stayed the same with age across a number of parameters. Increase in macrophage count or infiltration most commonly occurred in BMDMs (n = 5), peritoneum, adipose and liver (n = 4). Decrease in macrophage count or infiltration most commonly occurred in peritoneal (n = 6), BMDMs and muscle (n = 5), and wounds (n = 2). The proinflammatory (M1) macrophage markers, including CD11c, iNOS, MHC-II and CD80, were more frequently upregulated with age (n = 17), compared with downregulated (n = 3) and no change (n = 2). Increases in M1 macrophage populations were more often seen in wounds (n = 4), adipose tissue (n = 4) and alveoli (n = 3) and were also commonly reported in BMDM populations (n = 3). In contrast, alternatively activated (M2) macrophages were more commonly increased (n = 12) or decreased (n = 11) with age compared with no change (n = 7). The increased M2 population was more frequently seen in peritoneal (n = 3) hepatic (n = 2) or BMDMs (n = 2), whereas a decreased M2 population was most common in the muscles (n = 4) or peritoneum (n = 3). No change in M2 macrophage populations was less common, with two publications finding this in adipose tissue macrophages. We speculate that this lack of overall trend for age-related M2 macrophage counts may reflect the highly diverse phenotypes, with distinct roles in specific tissues or processes, of alternatively activated macrophages, that are often simplistically grouped together. Finally, IBA-1+ macrophages, IBA-1 being a marker of macrophage activation, were more frequently increased with age, with six out of seven studies, two different species and six different tissue macrophage populations demonstrating this expansion.

#### 3.2.2 Mediator release

There are a plethora of cytokines, chemokines and other soluble mediators released by macrophages. This is normally dependent on the tissue environment or polarisation state and governs the inflammatory phenotype of the surroundings (20), as well as the response to insult with ROS having key roles in antimicrobial activity and cellular differentiation (31). ROS such as superoxide has been linked to ageing, whereby increased catalysis to H2O2 has been associated with extended longevity in Drosophila (32). Considering the role of macrophages in inflammaging, much of the literature has assessed the effect of age on inflammatory mediator release. Data were extracted from publications that analysed soluble mediator release as a function of age and grouped according to whether an increase, decrease or no change was reported. These findings are summarised in **Figure 4B** as well as **Supplementary Table 2**, which again highlights the tissue and species of interest, as well as the environment of the cells assessed. TNF was evidently the most commonly assessed cytokine in ageing, most often being associated with reduced production. Most often, this decrease with age was measured after LPS and IFN-γ-stimulation (n = 15, **Figure 4C**), used to model inflammation *in vitro*, primarily in peritoneal and splenic macrophages. TNF secretion increase with age was most commonly found in BMDMs (n = 4), both in basal or inflammatory conditions. IL-6 and IL-1β were also commonly measured and were most often reported to be decreased with age (69% and 64% of reporting papers, respectively). These were often tested in conjunction with TNF; IL-6 decrease with age was also most commonly found as a response to LPS stimulation (**Figure 4D**). Anti-inflammatory or restorative (M2) macrophages were much less frequently assessed, with IL-10 being the predominantly analysed cytokine that is involved in inflammation resolution. IL-10 was most commonly increased with age, also more often being assessed in an inflammatory setting. ROS production was most commonly increased with age, including nitrogen oxide, free oxygen radicals and hydrogen peroxide.

#### 3.2.3 Phagocytosis, chemotaxis and pathogen recognition and killing

Phagocytosis is a core function of the macrophage, critically important in initiating the immune response and rapid pathogen clearance (33). A total of 20 papers addressed changes in phagocytosis with age; these were collated according to direction of change with age, be that upregulation, downregulation or no change, and then by macrophage type and species. The majority of these publications analysed peritoneal macrophages and C57BL/6 mice. Overall, 11 of the publications found a reduction in phagocytosis with age, seven found no change and two found phagocytosis to be increased with age (**Table 1**).

**Table 1:** Findings from publications assessing changes in phagocytosis with age, grouped by direction of change.

Direction of change with age	Macrophage type	Species	Additional factors	Citation
Upregulated (2)	Peritoneal (1)	BALB/C mice	Measured by phagocytic index, however downregulated compared with young with addition of NPY	(116)
	Pulmonary (1)	C57BL/6 mice	*M. tb* infection of cells	(117)
Downregulated (11)	Alveolar (2)	C57BL/6 mice (2)	Internalisation of *E. coli* particles	(33)
			Looked at percentage of cells phagocytosing latex beads	(72)
	BMDM (1)	C57BL/6 mice (1)	Assessed 4 days after reloading unloaded hindlimbs to assess aged skeletal muscle	(118)
	Peritoneal (8)	C57BL/6 mice (4)	Phagocytic activity of yeast cells	(119)
			Peritoneum-resident M1-like macrophages	(41)
			Fluorescent particle uptake by number of cells and particles	(120)
			Binding of IgG-5RBC and addition of protein diet	(121)
		ICR/CD1 mice (1)	Assessed long-lived mice and found positive correlation between achieved lifespan and phagocytic ability	(35)
		AO rats (1)	Zymosan, stimulation (LPS, GM-CSF, IL-4) enhanced phagocytosis in young but not old	(122)
		Swiss albino mice (1)	Percentage of phagocytes and phagocytic index of yeast cells	(123)
		BALB/C mice (1)	Without stimulation and with addition of NPY	(36)
	Microglia (1)	C57BL/6 mice	Rate of uptake of YG-beads over seven minutes	(124)
No change (7)	BMDM (3)	C57BL/6 mice (2)	Assessed many different treatments e.g., LPS/IFNγ	(125)
			Fluorescent particle uptake at different concentrations by number of cells and particles	(120)
		BALB/C mice (1)	Uptake of *S. pyogenes*	(126)
	Peritoneal (2)	Albino rat (1)	Percentage of phagocytosis in control, TG and TB samples	(127)
		A/J mice (1)	CdCO3 and latex particles	(128)
	MDM (1)	Human	*S. pneumoniae* binding and phagocytosis	(44)
	Hepatic (1)	C57BL/6 mice	FITC-labelled *E. coli* particles assessed in M1 and M2	(129)

BMDM, bone-marrow derived macrophage; MDM, monocyte-derived macrophage; NPY, Neuropeptide Y; M. tb, Mycobacterium tuberculosis; SRBC, Sheep red blood cells; LPS,Lipopolysaccharide; GM-CSF, Granulocyte macrophage colony stimulating factor; IL-4, Interleukin 4; YG, Yellow green; IFN, Interferon; TG, Thioglycolate; TB, Tumour cell implantation.

In order to successfully clear pathogens from tissues, macrophages also need to be able to migrate towards them, recognise them and kill them, be that via degradation in the phagolysosome or through recruitment of other immune cells (34). Looking at the chemotactic ability of aged macrophages, it was found, through assessment of macrophages in peritoneal suspension, that long-lived animals had overall better immune function, including phagocytosis and chemotaxis, than those that died at the previous age point studied. The very old and long-lived animals even had an increase in immune function compared with themselves at old age (35). Additionally, it was found that chemotaxis index increased with age (36), and that chemotaxis towards CCL2 was more robust in old bone marrow-derived macrophages (BMDMs) from C57BL/6 mice, enhanced further by sFASL (37). In contrast, chemotaxis was found to decrease with age in the final two publications, one assessing white adipose tissue in burns (38), the other looking at exercise, with aged controls having significantly less capacity for chemotaxis than young (39).

Pathogen recognition was a focus for very few papers, likely because of the complexity of the process, involving many different receptors and signalling pathways (40). However, it was suggested that old peritoneal macrophages had reduced ability to recognise pathogen associated molecular patterns (PAMPs) and signal through toll-like receptors, evidenced by reduced cytokine secretion (41). Macrophage killing ability was assessed by seven papers despite this being a less common function of these cells. In two studies, it was found that old BMDMs were less effective at clearing *S. pneumoniae* than young counterparts (42, 43). The same conclusion was drawn for peritoneal macrophages (43), as well as human monocyte derived macrophages (MDMs) (44). *C. albicans* killing was also found to be less effective with age, this time in peritoneal macrophages isolated from C57BL/6 mice (45). Conversely, the parasite *Leishmania* was found to be effectively killed by both young and old BMDMs (46), as well as *C. burnetti* bacteria by peritoneal macrophages (47). Finally, old peritoneal macrophages were found to be more able to resist HSV-1 than young counterparts (48).

#### 3.2.4 Morphology

Macrophage morphology as a function of age was assessed in five publications. Peritoneal macrophages isolated from old C57BL/6 mice were found to be larger and elongated with irregular morphology as compared to those from young mice (49). Macrophages isolated from the lateral wall of human cochlea from old donors appeared to have larger cytoplasmic volume and fewer projections than those from young donors (50). In the ageing cochlea of C57BL/6 mice, macrophages were enlarged and grainy with processes projecting towards adjacent macrophages. Giant macrophages with irregularly shaped nuclei were also found in the ageing cochlea but not in the young (51). Microglia morphology was assessed in two papers: in C57BL/6 mice, old microglia had less convoluted processes and accumulation of lipofuscin when compared with young (52). Conversely, those isolated from old F433 rats appeared to be more activated with amoeboid-like morphology (53).

#### 3.2.5 Gene and protein expression

Together, gene and protein expression have been the main focus in the literature relating to macrophage ageing to date. They are also crucially relevant when looking for biomarkers and potential therapeutic targets and were therefore collated into the MAIC in the following section.

### 3.3 Meta-analysis of macrophage ageing by information content

MAIC is a method of aggregating data from heterogenous sources without reliance on raw values or statistical quality scores. Each entity’s information content is quantified by measuring the number of times that it is mentioned across a body of literature. Therefore, the more an entity is mentioned throughout a literature pool, the higher the score is, indicating a strong association with the literature pool. The MAIC algorithm also includes a weighting function, giving a higher score to entities that have been assessed by multiple different methods (28).

We used the MAIC algorithm to assess the genes and proteins most associated with macrophage ageing, compiling 5 separate datafiles (as described in section 2.4) from the same set of 122 articles that had passed the inclusion criteria. These files were as follows:

1. All entities: 127 sets of genes and proteins
2. Upregulated Genes: 29 sets of genes demonstrating macrophage-specific upregulation with age in at least one experimental result
3. Downregulated Genes: 31 sets of genes demonstrating macrophage-specific downregulation with age in at least one experimental result
4. Upregulated Proteins: 50 sets of proteins demonstrating macrophage-specific upregulation with age in at least one experimental result
5. Downregulated Proteins: 53 sets of proteins demonstrating macrophage-specific downregulation with age in at least one experimental result

#### 3.3.1 MAIC for all entities

**Figure 5:**
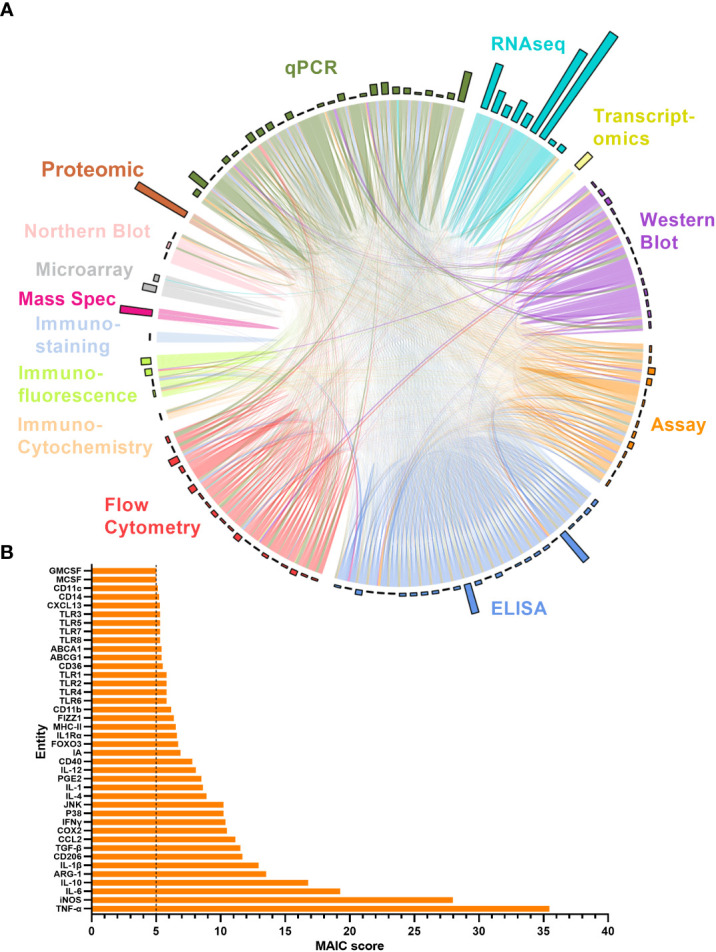
MAIC analysis results for entities associated with the ageing macrophage. **(A)** Outputs from the MAIC carried out on data for both genes and proteins across the literature were processed in R to produce a chord diagram where links (chords) between publications (segments) represent the sharing of a gene/protein. Publications are coloured (and grouped into sectors) by the methodology used to explore the entity. Chords stretching between publications differing in methodology are coloured by the more dominant scoring publication. The circle outer bars represent the total entities collected from each dataset/sector. **(B)** Top MAIC scoring entities across the literature with a cut-off score of 5.

The MAIC for all entities produced an overview of the level of representation of all genes and proteins explored in the literature in relation to ageing macrophages. The higher the MAIC score, the higher the level of representation across varying experimental methods throughout the literature (**Figure 5**). A total of 643 entities were outputted from the MAIC, with scores ranging from 1.0 to 35.5. The level of information content produced by each method is shown in **Figure 5A** corresponding to the magnitude of the respective coloured sectors on the chord diagram. This analysis found qPCR, ELISA, and flow cytometry to be the most frequently used methods for gene and protein expression assessment in this context. The outer circle bars represent the number of entities explored in the respective dataset, clearly showing that the majority of publications only recorded expression data for one or two entities per method used; RNA sequencing yielded the most data per publication. **Figure 5B** shows the calculated MAIC scores for each entity (gene or protein), with a minimum cut-off count of five. Here, TNF and iNOS were the most highly documented and investigated entities, with a score of 35.5 and 28, respectively.

#### 3.3.2 MAIC for gene and protein changes with age

The results of the “all entities MAIC” provides a quantitative evaluation for the level exploration of genes and proteins, as well as a comparative evaluation on the methodologies employed. However, this provided little information on the direction of change for these entities with age. A series of 4 more MAICs were designed to explore the direction of change for both genes (RNA) and proteins. As described in Section 2.4, any genes with results showing upregulation with age were collated into an MAIC input for upregulated genes, and the same applied for downregulated genes and up- and downregulated proteins.

**Figure 6:**
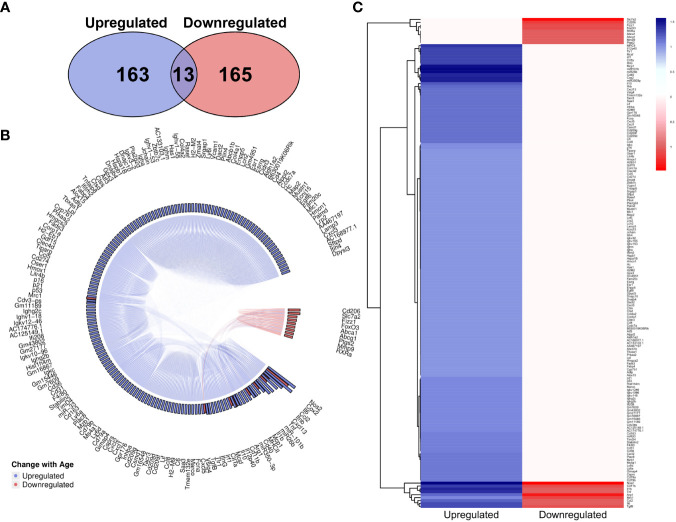
MAIC analysis results for changes in gene expression associated with macrophage ageing. **(A)** Number of genes observed in the literature according to direction of change with age. **(B)** Outputs from both the genes downregulated and genes upregulated MAICs were combined and processed with R into a chord diagram where links (chords) between genes (segments) represent cohabitation of the same dataset. Genes are coloured (and grouped into 2 sectors) by the dominant expression change with age. This is determined by whichever regulation shows a higher MAIC score and is portrayed in the outer ring of the circle by bars correlating with MAIC score. Thus, in the case of up and down regulation observed with age in the literature, both red and blue bars are shown with the larger bar governing the colour of chords stemming from that gene. **(C)** Mean MAIC scores for all genes, colour coded to match the documented change in expression with age.

The overall number of genes identified as upregulated and downregulated with age in the literature was highly comparable (**Figure 6A**), however there is a clear dominance of upregulated genes in the MAIC (**Figures 6B, C**), with the majority of downregulated genes having an MAIC score of only one (appearing in only one publication), putting them below the cut-off threshold. Overall, 159 genes were found to be upregulated with age and 18 were downregulated with age, according to MAIC score. Furthermore, 13 genes had an MAIC score for both upregulation and downregulation with age, indicating variation within the literature, but these scores were also higher for upregulation (**Figure 6B**).

**Figure 7:**
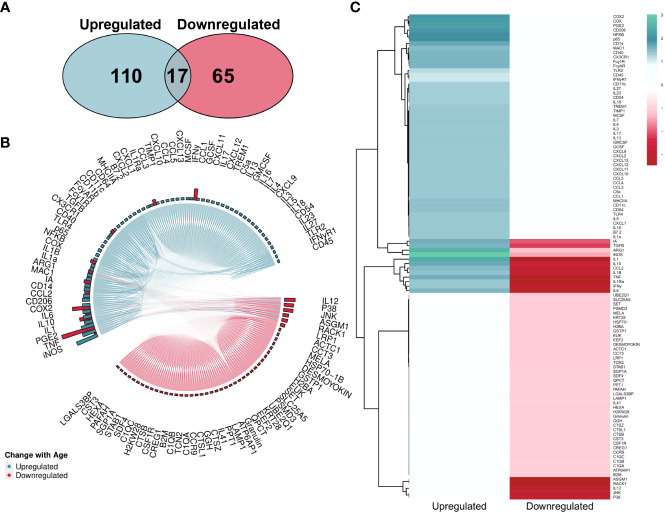
MAIC analysis results for changes in protein abundance associated with macrophage ageing. **(A)** Number of proteins observed in the literature according to direction of change with age. **(B)** Outputs from both the Proteins Downregulated and Proteins Upregulated MAICs were combined and processed with R into a chord diagram where links (chords) between proteins (segments) represent cohabitation of the same dataset. Proteins are coloured (and grouped into 2 sectors) by the dominant abundance change with age. This is determined by whichever regulation shows a higher MAIC score, shown in the outer ring of the circle by bars correlating with MAIC score. Thus, in the case of up and down regulation observed with age in the literature, both pink and green bars are shown with the larger bar governing the colour of chords stemming from that protein. **(C)** Mean MAIC scores for all proteins, colour coded to match the documented change in abundance with age.

The overall number of proteins identified within the literature was skewed towards upregulation (**Figure 7A**). However, MAIC scores were more evenly spread across both directions (**Figures 7B, C**) than that of the gene entities. This was due to the MAIC score for many upregulated proteins falling below the cut-off threshold. Similarly to that of genes, 12 proteins had scores in both directions of change with age, however in this instance scores were higher for downregulation (**Figure 7B**).

## 4 Discussion

Macrophages are a diverse and plastic cell type; their phenotypes can be divided into many different subcategories. Tissue populations often have unique ontogenies and differ widely from infiltrating populations. Local tissue environment also has a large impact on the way the macrophage functions (54–57). Partly as a consequence of this complexity, the literature has remained fragmented, in most cases, focussing on single models or tissues when investigating macrophage function. However, the ‘spectrum model’ is a recent major conceptual advance in our understanding of macrophage biology, highlighting the need to refer to macrophages by the stimulating agent/environmental cues controlling their function (58, 59). This review set out to consolidate published knowledge in the field, and although some conclusions can be drawn, clear gaps in the field have emerged, including the lack of functional work such as phagocytosis, being assessed in aged macrophages from different environmental settings.

Further to the sparsity of publications assessing age-related functional decline in the macrophage, it was surprising to find so few acknowledging the association between macrophages and senescence. Macrophages secrete many factors associated with the senescence-associated secretory phenotype (SASP) (20, 60), have been shown to undergo senescence *in vitro*, in response to ionizing radiation (60) or by high glucose exposure (61), and have a well understood role in clearance of senescent cells from their surroundings (62). There remains a lack of confidence as to whether non-replication competent cells such as macrophages can be senescent, as well as a lack of association between macrophage age and ability to respond to SASP or to clear senescent cells. One study by Hall et al. has found accumulation of senescent cells with age that appear to be a subpopulation of macrophages due to their removal with clodronate treatment (63). Further publications have also found increases in SA-beta-gal positive macrophages with age (49, 64). Finally, Kumar et al. did find that senescence increased in both young and old macrophage populations in the presence of conditioned media from senescent cells, which was rescued when media was switched to that from proliferating cells (49). A few studies have looked at how macrophages contribute to longevity with interesting results, finding long-lived animals have better overall immune function, including macrophage phagocytosis and chemotaxis, highlighting the need for properly functioning macrophages in healthy ageing (35). Dimitrivijec et al. also assessed long-lived animals and found long-lived rats had increased IL-10 expression and reduced TNF secretion as compared to old, highlighting the importance of balanced, macrophage-dependent inflammation in healthy ageing (65).

Macrophage cytokine release is a critical component of immunity, directing both initiation and resolution of inflammation. The three most commonly assessed cytokines in the macrophage ageing literature, TNF, IL-6 and IL-1β, are inflammatory cytokines that macrophages release in response to insult in order to recruit other immune cells such as leukocytes, to clear the insult through Th1 responses and induction of B cell proliferation and antibody production (66–68). Overall, the literature found expression of these cytokines to be decreased in aged compared with young macrophages in a pro-inflammatory setting. If secretion of these cytokines is decreased with age it likely indicates a lack of responsiveness to stimuli (69), so that macrophages are not mounting a full and effective inflammatory response. Although this may also have beneficial effects against some ARDs (70), the many signalling pathways involved have not been fully assessed in macrophages alone and an ageing setting. Frequently, when production of these pro-inflammatory cytokines was found to be increased with age, this was in a resting macrophage state, corresponding to a higher baseline inflammation with age, and indicating presence of low-level inflammation that could be contributing to many ARDs (71). It should be noted that there were many more data looking at an inflammatory setting than anti-inflammatory/restorative, and this is likely due to the ease of modelling an infection, most commonly stimulating with LPS and IFN-γ (72). This is compared with the complex nature of tissue restoration, with many different components and macrophage subtypes falling into this category (73, 74). Macrophages can be stimulated with IL-4, to create a wound healing phenotype, immune complexes to create a regulatory phenotype that upregulates IL-10 and many more reviewed elsewhere (73–76). Overall, only IL-10 secretion was well characterised across the literature, with an increase with age in an inflammatory setting being the overall conclusion. This could indicate immune system dysfunction, where resolution is occurring during the inflammatory response, with Chelvarajan et al. finding an increase in IL-10 in LPS-stimulated aged macrophages contributing to a decrease in ability to produce proinflammatory cytokines (77); however, this complex and dynamic system is still not fully understood. Zhao et al. linked an increase in IL-10 to neovascularisation, making age-related macular degeneration more aggressive (37). Alongside IL-10, overproduction of ROS appeared commonplace in ageing (78, 79), which could lead to excess tissue and DNA damage through oxidative inflammatory stress (78), cytotoxicity and abnormal growth of cancer cells (80).

It is widely thought that overall, tissue macrophage numbers do not change with age (81–83), although expansion of infiltrating macrophages has been reported (84). Each of the pan macrophage markers and leukocyte specific markers assessed in the literature showed much heterogeneity in expression; F4/80 has a comparable number of papers that find expression increase (n = 13), decrease (n = 10) and no change (n = 10). This may relate to the fact that many different tissue types have been assessed, with decrease in F4/80-expressing macrophages occurring more frequently in the peritoneum (45, 64, 85) and increase most commonly in infiltrating BMDMs (86, 87). On the other hand, human CD68^+^ macrophages were more consistently present with age (no change: n = 3, increase: n = 1, decrease: n = 1) and rat ED-1^+^ macrophages, the homolog of CD68, were more frequent with age (increase n = 6, decrease n = 1, no change n =3). It was more common that macrophages expressing M1 markers were increased with age, while macrophages expressing M2 markers showed a trend towards being decreased with age. This could explain the heterogeneity in pan-macrophage markers as ageing is not leading to an expansion of macrophages so much as a change in marker expression (87–92). One commonality in the literature was an overall decrease in pan-macrophage marker expression and increase in M1 marker expression in wounds (93–95), supporting the theory that macrophage populations are shifting rather than expanding. This could potentially advance the field in improving recovery time to wounds in older individuals. Another consistency in the literature that could be potentially exploited was that IBA-1^+^ macrophage populations increased with age (96–99). This was across a number of different macrophage subtypes assessed, highlighting its homogeneity in the ageing macrophage and thus could be further explored as a potential biomarker or therapeutic target for macrophage ageing.

In terms of functional changes with age, where phagocytosis was assessed, the majority of publications found it to be reduced with age. This likely contributes to reduction in ability to respond to infection with age, as reduced phagocytosis would reduce pathogen recognition and antigen presentation as well as pathogen clearance, halting the adaptive immune response (100, 101). There were also instances where phagocytosis was upregulated with age, in particular peritoneal macrophages showed more variable phagocytosis with age, as different publications cam to different conclusions. In reality, upregulated phagocytosis with age may not correlate with effective processing of phagocytosed contents or presenting antigens to the adaptive immune system, and changes with age in either direction could indicate immune dysfunction (100, 101). Pathogen recognition and antigen presentation are much more difficult to quantify with age; antigen presentation requires more than one cell and at least three different receptor binding events (102). It has not yet been characterised in ageing more extensively than reduced cytokine secretion after PAMP recognition (41). Overall chemotaxis was found to be increased in two publications (36, 37) and decreased in three with age (35, 38, 39), although changes with age were found in each instance supporting the notion of immune system dysfunction with age. There is a clear need for more research into macrophage functional decline with age and the mechanisms behind this. Morphological changes were assessed by five papers and commonly, it was found that macrophage size increased with age, as well as projections (49–51). This suggests an observably distinct phenotype with age compared with healthy young counterparts.

The MAIC was conducted to better define age-related changes of genes and proteins by quantifying the observations in the literature. MAIC is estimative in its statistical power, however the results gained are highly informative if the assumption is made that the literature included is unbiased and the level of investigation of each factor is directly correlated with its association with the ageing macrophage. For this reason the method used is included alongside entity, since RNA sequencing and other high throughput methods have much greater breadth and therefore bias towards no change with age compared with that of high specificity, candidate-based methods such as qPCR or western blot. Therefore, entities sharing the same changes with age across these different methods score much more highly in the MAIC, reflecting the increased likelihood that these results represent a true change with age. Because of the breadth and lack of specificity in high throughput methods, entities with MAIC scores of 1 were omitted, also indicative that these entities have no shared information content, assessed by only one method.

Cytokines TNF, IL-6, IL-10 and IL-1β were all among the top 10 most assessed entities, as well as macrophage markers iNOS, Arg-1 and CD206. These are of course among the most well-known genes and proteins associated with the macrophage, alongside several of the other entities with a score of 5 or higher (**Figure 5**). It is interesting therefore to recognise that some of the less candidate-based entities may prove to be equally important as markers and functionally in macrophage ageing, This systematic MAIC provides a new tool to consolidate these findings and identify previously overlooked potential biomarkers or therapeutic targets. ABCA1 and ABCG1 belong to the ATP-binding cassette family and function together as a cholesterol efflux pump for cellular lipid removal (103). These proteins have been shown to be relatively highly expressed in the macrophage and dysregulation of these genes is known to be associated with dyslipidaemia which may be relevant in ARDs such as atherosclerosis (104). FIZZ1, also known as RETNLB or resistin like beta, enables adipose hormone activity (105) and is involved in IL-4 signalling (106). It has also been implicated in the development of atherosclerosis (107). Each of these genes could prove interesting to further explore in the ageing macrophage setting.

Overall, 159 genes were found to be upregulated and 18 downregulated with age across at least 2 methods or publications, while 13 genes were found to be both up- and down-regulated (**Figure 6**). These 12 genes constitute many of the entities with the highest overall MAIC scores, probably indicating the amount of data relating to them is dictating the overall findings. It should also be noted that the MAIC presented here does not take into account the tissue environment of the macrophage. Discussed previously in relation to changes in secretion of pro-inflammatory cytokines and expression of different macrophage markers, environmental cues may potentially be causing the changes in regulation reported. Again, it is unsurprising that there are many genes in this list that are well established in macrophage function, including genes encoding macrophage-specific markers, cytokines and chemokines. For example, MRC1/CD206 is a pattern recognition receptor involved in pathogen sensing that modulates macrophage polarisation (108). It was found to be both upregulated and downregulated with age, whereas MCP-1 is a monocyte-specific chemokine that was consistently upregulated with age. Of note, CD40 and COX2 were present in the MAIC for genes and had high overall MAIC scores, so will be interesting to research further in the context of macrophage ageing. CD40 is a receptor on antigen presenting cells for binding with T and B cells and bridging the innate and adaptive immune system (109). As it forms a crucial part of the immune system its dysregulation may very well lead to age-related immune dysfunction. COX2 is a key enzyme in prostaglandin synthesis, with a key role in inflammation (110). Existing literature has found this to be upregulated with age and this too could be very interesting to further explore in relation to chronic inflammaging.

For the protein MAIC, 62 were upregulated with age and 58 downregulated with a significant MAIC score. 12 proteins were both up- and down-regulated across the literature. This included upregulation of many cytokines and chemokines, as well as CD40 and COX2, consistent with that seen in the gene MAIC. JNK and p38 were both downregulated with age. These are key effectors in MAPK signalling pathways that regulate many cellular processes from proliferation to apoptosis and inflammation (111). It has been reported that this downregulation seen with age leads to defective TLR-mediated signalling (112), phagocytosis and overall immune function (113). Receptors CD206 and MAC-1 were upregulated with age. These receptors are involved in pathogen sensing, as well as CD206 being an anti-inflammatory macrophage marker. RACK1 was also found to be downregulated across the literature. This protein has been shown to play a role in M2/M1 macrophage ratio and so its dysregulation could shift macrophages towards an inflammatory phenotype (114). Overall, there are a number of consistently dysregulated proteins, as well as genes, that could form a panel for assessing biological age of macrophages. An important next step to this review could therefore be to assess these genes and proteins thoroughly across different species and tissues to validate this list and provide a platform for the discovery of the novel biomarkers in the age-related context.

There is now a plethora of literature relating to macrophage ageing, whilst there are still large gaps in the field, which are limited by the lack of functional work that has so far been completed on different macrophage types in an ageing setting. Of the 240 publications included, only 20 investigated phagocytosis, usually the most well-documented function of the macrophage and only 5 assessed chemotaxis, crucially important in infiltration or surveillance of the tissue (115). Gene and protein expression were much better studied, with as many as 9 separate RNA sequencing datasets, two microarray datasets and a further proteomic dataset contributing to the MAIC, likely accounting for genes and proteins that have been previously under analysed. Data sharing is now crucially important to validate and expand this list of entities critical to the ageing macrophage phenotype; a starting point in biomarker recognition might be macrophage marker of activation IBA-1, for which a clear upregulation with age was seen across the literature. Additionally, many of the genes and proteins found in the MAIC to be consistently dysregulated, such as CD40, COX2, RACK1 and the ABC1 family could be added to produce a panel of entities crucial to the macrophage ageing phenotype.

## 5 Conclusion

By compiling the macrophage ageing literature to date, we can conclude that macrophages undergo functional decline with age, including reduced phagocytosis and chemotaxis, reduction in cytokine release in response to inflammatory stimuli and increased ROS production. Additionally, through our meta-analysis by information content we have found many genes, proteins, and soluble mediators that change with age (**Table 2**). Further assessment of these markers across different macrophage populations and environments could result in the identification of a consistent panel of age-related macrophage markers. Alongside functional decline of macrophages in response to age seen within this review, macrophage biological age could be further established using this panel of genes and proteins, which may in turn shed light on the pathways resulting in macrophage functional decline. Overall, this panel should serve as a useful resource in designing future studies assessing macrophage ageing.

**Table 2:** Dysregulated genes and proteins with age that have the highest information content from the meta-analysis.

Genes		Proteins	
Upregulated	Downregulated	Upregulated	Downregulated
MHC-II	CD206	COX2	ASGM1
MCSF	FIZZ1	PGE2	RACK1
MCP1	ABCA1	CD206	IL12
CD40	ABCG1	NFKB	JNK
COX2	MMP9	CD40	P38
IL-10	FOXO3	MAC1	LRP1

A starting point for further analysis in order to create a panel of age-related macrophage markers.

## Data availability statement

The original contributions presented in the study are included in the article/**Supplementary Material**. Further inquiries can be directed to the corresponding author.

## Author contributions

CM, HP, HW and EK-T designed the review. CM and HP conducted the search, screening and data extraction and analysis. All authors contributed to writing and editing of the manuscript. All authors read and approved the final manuscript. All authors contributed to the article and approved the submitted version.
