# Supplementary material for: Markers of the ageing macrophage: a systematic review and meta-analysis
Source: Front Immunol. 2023 Jul 13;14:1222308. doi: 10.3389/fimmu.2023.1222308 (PMC10373068; doi:10.3389/fimmu.2023.1222308)
Supplement: Supplementary file 1 [file DataSheet_1.docx]

**Supplementary material**

**Supplementary Table 1: Input table for MAIC. Number followed by letter indicates the publication and then different method used**

| **Method** | **Publication** | **Entity type** | **Entity names** |
| --- | --- | --- | --- |
| qPCR | [1a] | Gene | CD206, FIZZ1, TFGB, TNF, IL6, NOS2, FOXO3 |
| Western Blot | [1b] | Protein | FOXO3 |
| RNAseq | [9a] | Gene | IL4RA, IL13RA1, ARG1, RETNLA, IL13RA2, NOS1, NOS2 |
| Flow Cytometry | [9b] | Protein | MHCII, Ki67, FIZZ1 |
| Flow Cytometry | [11a] | Protein | CD11b, CD80 |
| ELISA | [11b] | Protein | IL1B, IL6, TNF, IL10 |
| qPCR | [11c] | Gene | ARG1, MRC1, MSR1, P53, P21, P16 |
| ELISA | [15a] | Protein | TNF, IL1, IL12 |
| ELISA | [15b] | Protein | TNF, IL1, IL12 |
| ELISA | [15c] | Protein | TNF, IL1, IL12 |
| qPCR | [24] | Gene | ARG1, NOS2, SLC7A2 |
| qPCR | [32] | Gene | TNF, NOS2, ARG1, CD206, IL1RA |
| Flow Cytometry | [33a] | Protein | CD36 |
| qPCR | [33b] | Gene | Fads2, Fdft1, Fdps, Scd1, Cyp11a1, Cyp27a1, Hadhb, Nr0b2, Nr1h3, Lta4h, Alox5ap, Alka1, Apof, Slc16a6, Slc27a1, Slc27a3, Stard4, Cxcl16, Olr1, Pcsk9, Stab2, p16, Fads3, Hmgcs2, Prkaa2, Cyp7b1, Lpl, Tbxas1, Adfp, Alox15, Fabp4 |
| Transcriptomic | [33c] | Gene | Scd2, Dhcr24, Acsl3, Ceacam19, Idi1, Clec5a, Mir15a, Sgle, Cyp51, Csprs, Gimap4, Cd79a, Ly6d, Ms4a1, Cd79b, Mzb1, Cxcl13, Plac8, Ly6a |
| Mass Spec | [41] | Protein | QPCT, Granulin, ATP6AP1, LAMP1, PPT1, IL-41, CTSZ, GGH, CTSL1, CCR9, C1QA, TCN2, C1QB, LRP1, B2M, CREG1, CSF1R, CTSB, H2KW28, C1QC, SDF4, STAB1, SGP1A, PAFAH, HEXA, CST3, LGALS3BP, PRRG2, FBN1, COL1A1, POSTN, PAI1, COL1A2 |
| ELISA | [41] | Protein | LRP1 |
| Northern Blot | [55] | Gene | NOS2 |
| Assay | [61] | Protein | PGE2, COX |
| Assay | [71] | Protein | PGE2, COX |
| Assay | [85] | Protein | TNF |
| Assay | [96] | Protein | TNF, ASGM1, IA |
| ELISA | [99] | Protein | IL1, TNF |
| Flow Cytometry | [110a] | Protein | CCR7, CD163 |
| Assay | [110b] | Protein | ARG1, NOS2 |
| ELISA | [110c] | Protein | IL1B, IL6, TGFB, IL10 |
| qPCR | [127a] | Gene | CCL2, IFNB, TNF, IL12p40, IL10, MCSF, CD11b, COX2, MHCIIAa, CIITA, IRF1, IFNg, TLR1, IL1B, GMCSF, CD11c, CD206, TLR2, TLR4, TLR6 |
| Flow Cytometry | [127b] | Protein | CD11b, CD11c, CD206, CD64, MHCIIA |
| Flow Cytometry | [129a] | Protein | CD14, TLR4 |
| ELISA | [129b] | Protein | TNF, IL10 |
| Assay | [129c] | Protein | ARG1, iNOS |
| Assay | [133a] | Protein | NFKB, p65, PGE2, COX2, CREB, AP-1, IKBa |
| Western Blot | [133b] | Gene | COX2, NOS2 |
| Assay | [134] | Protein | ERK, JNK, P38, PGE2, COX2 |
| Western Blot | [137] | Protein | STAT1B, STAT1A, STAT1Bp, STAT1Ap |
| qPCR | [137] | Gene | STAT1 |
| Northern Blot | [147] | Gene | hsp70 |
| ELISA | [149] | Protein | TNF, IL6 |
| Western Blot | [150a] | Protein | TNF, RACK1, CD14, PKCA, PKCBII |
| qPCR | [150b] | Gene | TNF |
| ELISA | [156a] | Protein | TNF, IL1RA, IL6, IL10, CCL2, CCL3, CCL4, CCL5, CXCL1, CXCL2, CXCL10, TIMP1, CXCL13, GCSF, GMCSF, CCL1, CD54, IFNY, IL1A, IL1B, IL16, IL17, IL23, IL27, IP10, MCSF, CXCL12, TREM1, IL2, IL3, IL5, IL7, IL13, CXCL11, CCL1, CXCL9, CCL17, C5A |
| RNAseq | [156b] | Gene | Gm42427, Wdfy1, Tmem181bp, Dynlt1b, Cd59a, Sel1l3, Tmem181bp, Gm42031, Gm43305, Xlr, Rab4a, Nbea, AC157515.1, Gm42742, C2, Cfap69, Steap2, Stc1, Vegfc, Steap1, Fgf23, Hist1h4n, Gm10359, Saa1, Slc6a14, 1700028P14P, Lhb, mt-Nd4l, Gm14176, Gm5776, Emx2, Gm7609, Gm15446, Igkc, Gm16867, Hist1h4m, Ighg2b, Igkv10-96, Gm27177, Gm43802, Ifi208, AC125149.1, AC174776.1, Igkv12-46, Ighv1-18, Ighg2c, Ifi208, Ifnb1, Gm11189, Cdv3-ps |
| RNAseq | [157] | Gene | Vsig4, Cxcl13, Cxcl5, Fcna, Marco, Tmem132e, Saa3, C6, H2-M9, Ccl8, Lif, Cd209f, Cd209d, Tacr3, Gm16548, Csmd1, Cd209g, Inhba, Gpr176, Cxcl1, Cxcl2, Il6, Ccl2 |
| qPCR | [158a] | Gene | Cox2, RXRa, Abca1, Abcg1, LXRa, LXRb |
| Western Blot | [158b] | Protein | COX2, RXRa |
| ELISA | [158c] | Protein | PGE2 |
| qPCR | [167] | Gene | Cd11b, Cd206, TGFB |
| Proteomic | [183] | Protein | MYO1F, RARS, DPYS11, PIK3AP1, HUWE1, TPP1, NAPRT, PPAT, SF3B2, MARS, PGAM2, ACTG1, F1NC, CAPG, TUBA1A, ACTA1, YWHAZ, CAP1, ANXA5, F1NA, CTSD, H2-D1, HSC70, TIP-B1, FGH, GM2A, GPX1, ANXA6, CTSB, PRX, MDH2, ANXA2, UBA1, PGK, LCP1, ANXA1, HSP90AB1, ALDH-2, GRP78, HA1, LYZ, MOM2, VAT-1, RP14, ACTC1, CCT3, MELA, HSP70-1B, ARG1, DESMOYOKIN, GSTP1, H2BA, EUK, SET, SLC25A5, PSMD3, UBE2Q1, KRT28, EEF2 |
| Immuno Cytochemistry | [186a] | Protein | iNOS |
| qPCR | [186b] | Gene | Nos2 |
| qPCR | [188a] | Gene | TLR1, TLR2, TLR3, TLR4, TLR5, TLR6, TLR7, TLR8, TLR9 |
| Flow Cytometry | [188b] | Protein | TLR4 |
| ELISA | [188c] | Protein | TNF, IL6 |
| qPCR | [189a] | Gene | TNF, Nos2, CD40, CD11b, MHCII, Arg1, Mrc1 |
| Flow Cytometry | [189b] | Protein | IFNyR1, CD11b, CD45 |
| ELISA | [189c] | Protein | TNF |
| qPCR | [190a] | Gene | FoxO3, Cd206, TgfB, Fizz1, Nos2, Tnf, Il6 |
| Western Blot | [190b] | Protein | FOXO3 |
| Flow Cytometry | [194a] | Protein | IA |
| Western Blot | [194b] | Protein | IA |
| Northern Blot | [194c] | Gene | IaB, IeB, CIITA |
| ELISA | [196a] | Protein | TNF, IL6, P38, JNK |
| Western Blot | [196b] | Protein | P38, JNK |
| Flow Cytometry | [196c] | Protein | TLR4 |
| Flow Cytometry | [197] | Protein | TNF, IFNy, IL12, IL6, CCL2, IL10 |
| ELISA | [198a] | Protein | TNF |
| Assay | [198b] | Protein | PGI2 |
| qPCR | [198c] | Gene | TNF |
| ELISA | [201a] | Protein | TNF, IL1B, IL6, IL12 |
| Flow Cytometry | [201b] | Protein | TLR4, IL-6Ra |
| RNAseq | [206a] | Gene | Gm16867, Hist1h4m, 1700112E06Rik, Gm15446, Wdfy1, Tmem181b-ps, Dynlt1b, Marco, Csf2, Smo, Efcab7, Ptch1 |
| Western Blot | [206b] | Protein | WDFY1 |
| ELISA | [206c] | Protein | CXCL13, C5a, G-CSF, GM-CSF, CCL1, IFNy, IL1a, IL1B, IL3, IL4, IL7, IL13, IL18, IL17, CXCL10, CXCL11, CXCL9, CCL3, CXCL2, CXCL12, TIMP1, TREM1, CCL4, CD54, CCL5, TNF, IL6, IL1ra, MCSF, CCL4, IL23, IL27 |
| RNAseq | [207] | Gene | Vsig4, Cxcl13, Cxcl5, Fcna, Marco, Tmem132e, Saa3, C6, H2-M9, Ccl8, Lif, Cd209f, Cd209d, Tacr3, Gm16548, Csmd1, Cd209g, Inhba, Gpr176, Cxcl1, Cxcl2, Il6, Ccl2 |
| Assay | [211a] | Protein | iNOS, ARG1 |
| ELISA | [211b] | Protein | IL1a, IL10, TNF |
| Assay | [212] | Protein | TNF |
| qPCR | [216] | Gene | Reverba |
| ELISA | [226] | Protein | IL6 |
| Flow Cytometry | [230a] | Protein | TLR4, Marco |
| qPCR | [230b] | Gene | P16inka |
| Immunostaining | [230c] | Protein | BGalactosidase |
| Assay | [233a] | Protein | ARG1, iNOS |
| Flow Cytometry | [233b] | Protein | CD86, CD40, CD80, CD206 |
| ELISA | [245a] | Protein | TNF, IL1, IL6 |
| Assay | [245b] | Protein | TNF, IL1, IL6 |
| ELISA | [249a] | Protein | TNF, IL6, IL10 |
| Flow Cytometry | [249b] | Protein | TLR2, TLR4 |
| Western Blot | [249c] | Protein | p38, MAPK-APK-2 |
| RNAseq | [253] | Gene | Gm10260, Lilr4b, Hmox1, Oser1, Cd274, Ccl5, Tiparp, Clec4d, Gdf15, Cxcl2, H2-Eb1, Gm10036, Ly6e, Coro1a |
| Flow Cytometry | [254a] | Protein | Marco, TLR4 |
| qPCR | [254b] | Gene | Rac1, N-WASP |
| Western Blot | [254c] | Protein | RAC1, N-WASP |
| Western Blot | [260a] | Protein | p62, ATG5, LC3-II |
| ELISA | [260b] | Protein | TNF, IL1B, IL6 |
| Immuno flourescence | [260c] | Protein | iNOS, CD206 |
| qPCR | [260d] | Gene | Tnf, Il1b, Il6, iNOS, Mcp1, Mrc1, Arg1 |
| Microarray | [261] | Gene | miR-181c, miR-3068, miR-330, miR-5135, miR-10a, miR720, miR-29c, miR-714, miR-150, miR-195, miR-3273, miR-145, let-7d, miR-1247 |
| Immuno flourescence | [264] | Protein | TNF |
| ELISA | [268] | Protein | IL1B, IL6, TNF |
| qPCR | [274a] | Gene | miR-142-3p |
| ELISA | [274b] | Protein | IL6 |
| qPCR | [277a] | Gene | Cox2, miR-101b, miR26b |
| ELISA | [277b] | Protein | PGE2 |
| Western Blot | [280] | Protein | A20, CYLD |
| Western Blot | [281] | Protein | COX2, PGE2, COX1 |
| qPCR | [290a] | Gene | ABCA1, ABCG1, TNF, IL1B, PTGS2, CCL2, MMP9, TGFB, IL10, CD163, miR-33 |
| Western Blot | [290b] | Protein | ABCA1, ABCG1 |
| qPCR | [291] | Gene | TIMD4, Stabilin2, IL10, F4/80, CD31, CD36, ARG1, TNF, IL6, IL1B, IL4, CD68 |
| RNAseq | [301a] | Gene | miR10a, miR10b, miR130a, miR142, miR19a, miR19b-1, miR19b-2, miR210, miR29b-1, miR301, miR301b, miR340, miR106a, miR125a, miR125b-1, miR125b-2, miR199b, miR24-1, miR24-2, miR30a, miR30d, miR30e, miR331, miR339, miR99b, miR103-1, miR107, miR126, miR140, miR148a, miR150, miR15b, miR181a-1, miR181b-2, miR185, miR191, miR192, miR1944, miR23a, miR25, miR26b, miR532, miR92-2, miR92b, miRlet7i, miR100, miR139, miR181a-2, miR222, miR23b, miR26a-1, miR26a-2, miR27a, miR27b, miR30b, miR30c-1, miR30c-2, miR324, miR484, miR744, miR99a, miR101a, miR101b, miR103-2, miR16-1, miR16-2, miR34a, miR425, miR451, miR7-1, miR7-2, miR7b, miR93, miRete.bSep07, miRlet7c-1, miRlet7d, miR155, miR1839, miR199a-1, miR199b, miR20a, miR218-1, miR221, miR31, miR322, miRlet7a-1, miRlet7a-2, miRlet7c-2, miRlet7e, miRlet7f-1, mIrlet7f-2, miRlet7g, miR29a, miR146a, miR18a, miR146b, miR22, miR423, miR15a |
| qPCR | [301b] | Gene | miR146b, miR22, miR423, miR15a, miR29a, miR146a, miR18a |
| Microarray | [302] | Gene | miR143, miR145, miR150, miR195, miR720 |
| Immuno flourescence | [306] | Protein | FcuR, Fcy2AR, Fcy2BR, FceR, Fcy1r, FcaR, Ia, MAC1 |
| qPCR | [308] | Gene | Tnf, Il1b, Il6, Il12/p40, TgfB, Il10 |
| qPCR | [310] | Gene | miR350-3p, Il6 |
| Flow Cytometry | [327] | Protein | IL10, CD40, CX3CR1, TGFB, IFNy, IL4, TNF |
| qPCR | [328] | Gene | Stat5a, Stat5b |
| qPCR | [329] | Gene | iNOS, Il1B, Il6, MHCII, CD40, TNF |
| Immuno flourescence | [333] | Protein | C5b, C3bi, IgG1, C3b, IgG2a, IgG2b, IgG3, IgM, IgA, IgE |
| Flow Cytometry | [334a] | Protein | ED2 |
| ELISA | [334b] | Protein | TNF |
| RNAseq | [335] | Gene | Ifitm2, Tnfaip8, Ctsd, Ctss, Atf3, Gpx3, Dnajb4, Hspb1, Hspa1B, Dnajc10, Marco, Igkv3-2, Pla2g2d, Igha, Jchain, Ighv1-53, Zbtb7c, Igkc, AC133103.1, Ighm, Has1, Ighv1-55, Col4a2, Rasef, H2-M2, Zmat4, Srgap1, Ly6i, Vcam1, Dact2, Plin4, Abcb1b, Col4a1, Enpp5, Lcn2, Gm4951, Esr1, Esrrg, Cldn3, Aldh1a2, B630019K06Rik, Cd4, Alppl2, Ccdc7a, Hc, Map2, Mustn1, Kcnj15, Egfl6, Fam20c, Mlc1, Hmcn1, Palmd, Lrp2, AA467197, Lamp3, Cxcl3, AC168977.1, Sftpd, Itih4, Dpysl3, Egfem1, Ovol2, Adora3, Ikzf4, Inka1, Mterf2, Nr1d1, Zfp979, Penk, Itga6, A430106G13Rik, Btla, Esco2, Cenpk, Fam221a, Zbtb32, Clec4a1, Cenpw, Cbfa2t3, Ska1, Mir142b, Haao, Id3, E2f7, Cenpm, Rmi2, Fcho1, Ica1, Fam78b, Cdca7, Cd93, Fignl1, Cbx2, Nid2, Ptp4a3, Pclaf, Ccna2, Depdc1a, Myrip, Lrrc75a, Abi3, Fam83d, Fam92a, Uhrf1, C1qtnf12, Rad54b, Chek1, Sapcd2, Psat1, Deldc1b, Ifg1, Pdgfa, C3, Egfr, Rab27B, Cd36, Rab25, P2ry1, Sod3, Ccnt1, Csf1, Siglec1, Rab12, Hsf4, Csf2ra, Csf2rb, Csf1r, Il3ra, Jak2 |
| ELISA | [338a] | Protein | IL10, IL1B, IL6, IL12, TNF |
| Flow Cytometry | [338b] | Protein | MAC1, B7.2, TLR4, CD14 |
| Flow Cytometry | [339a] | Protein | TLR1, TLR2, TLR3, TLR4, TLR5, TLR6, TLR7, TLR8, TNF, IL6 |
| Assay | [339b] | Protein | TNF, IL6, IL1B, IFNy, IL10 |
| RNAseq | [341] | Gene | Ncan, Vcan, Cd44, Cspg4, Fibronectin |

**Supplementary Table 2 – Input table for genes upregulated MAIC analysis**

| **Method** | **Publications** | **Gene names** |
| --- | --- | --- |
| qPCR | [1] | Tnf, Nos2, Il6 |
| qPCR | [11] | Arg1, Mrc1, p53, p21, p16 |
| qPCR | [32] | Tnf |
| Transcriptomic | [33a] | Csprs, Gimap4, Cd79a, Ly6d, Ms4a1, Cd79b, Mzb1, Cxcl13, Plac8, Ly6a |
| qPCR | [33b] | p16, Fads3, Hmgcs2, Prkaa2, Cyp7b1, Lpl, Tbxas1, Adfp, Alox14, Fabp4 |
| qPCR | [127] | Ccl2, IfnB, Tnf, Il12p40, Il10, Mcsf, Cd11b, Cox2, MHCII, CIITa, Irf1, IfnG, Tlr1 |
| Western Blot | [133] | Nos2, Cox2 |
| RNAseq | [156] | Gm7609, Gm15446, Igkc, Gm16867, Hist1h4m, Ighg2b, Igkv10-96, Gm27177, Gm43802, Ifi208, AC125149.1, AC174776.1, Igkv12-46, Ighv1-18, Ighg2c, Ifi208, Ifnb1, Gm11189, Cdv3-ps |
| RNAseq | [157] | Vsig4, Cxcl13, Cxcl5, Fcna, Marco, Tmem132e, Saa3, C6, H2-M9, Ccl8, Lif, Cd209f, Cd209d, Tacr3, Gm16548, Csmd1, Cd209g, Inhba, Gpr176, Cxcl1, Cxcl2, Il6, Ccl2 |
| qPCR | [158] | Cox2 |
| qPCR | [186] | Nos2 |
| qPCR | [189] | Tnf, Nos2, Cd40, Cd11b, MHCII, Arg1 |
| qPCR | [190] | Nos2, Tnf, Il6 |
| qPCR | [198] | Tnf |
| RNAseq | [206] | Hist1h4m |
| RNAseq | [207] | Vsig4, Cxcl13, Cxcl5, Fcna, Marco, Tmem132e, Saa3, C6, H2-M9, Ccl8, Lif, Cd209f, Cd209d, Tacr3, Gm16548, Csmd1, Cd209g, Inhba, Gpr176, Cxcl1, Cxcl2, Il6, Ccl2 |
| qPCR | [230] | P16inka |
| RNAseq | [253] | Lilr4b, Hmox1, Oser1, Cd274, Ccl5, Tiparp, Clec4d, Gdf15, Cxcl2, H2-Eb1, Coro1a |
| qPCR | [260] | Tnf, Il1b, Il6, Nos2, Mcp1 |
| Microarray | [261] | miR-714, miR-150, miR-195, miR-3273, miR-145, Let-7d, miR-1247 |
| qPCR | [277] | Cox2, miR-101b, miR26b |
| qPCR | [290] | Il10, Cd163, miR-33 |
| qPCR | [291] | TimD4, Stabilin2, Il10, F4/80, Cd31, Cd36, Arg1 |
| Microarray | [302] | miR143, miR145, miR150, miR195 |
| qPCR | [308] | Tnf, Il1b, Il6, Il12p40, TgfB, Il10 |
| qPCR | [310] | miR350-3p, Il6 |
| qPCR | [329] | Nos2, Il1b, Il6, MHCII, Cd40 |
| RNAseq | [335] | Ifitm2, Tnfaip8, Ctsd, Ctss, Atf3, Gpx3, Dnajb4, Hspb1, Hspa1B, Dnajc10, Marco, Igkv3-2, Pla2g2d, Igha, Jchain, Ighv1-53, Zbtb7c, Igkc, AC133103.1, Ighm, Has1, Ighv1-55, Col4a2, Rasef, H2-M2, Zmat4, Srgap1, Ly6i, Vcam1, Dact2, Plin4, Abcb1b, Col4a1, Enpp5, Lcn2, Gm4951, Esr1, Esrrg, Cldn3, Aldh1a2, B630019K06Rik, Cd4, Alppl2, Ccdc7a, Hc, Map2, Mustn1, Kcnj15, Egfl6, Fam20c, Mlc1, Hmcn1, Palmd, Lrp2, AA467197, Lamp3, Cxcl3, AC168977.1, Sftpd, Itih4, Dpysl3 |
| RNAseq | [341] | Ncan, Vcan, Cd44, Cspg4, Fibronectin |

**Supplementary Table 3 – Input table for genes downregulated MAIC analysis**

| **Method** | **Publication** | **Gene names** |
| --- | --- | --- |
| qPCR | [1] | Cd206, Fizz1, TgfB |
| qPCR | [24] | Arg1, Nos2, Slc7a2 |
| qPCR | [32] | Nos2, Arg1, Cd206 |
| Transcriptomic | [33a] | Scd2, Dhcr24, Acsl3, Ceacam19, Idi1, Clec5a, Mir15a, Sgle, Cyp51 |
| qPCR | [33b] | Fads2, Fdft1, Fdps, Scd1, Cyp11a1, Cyp27a1, Hadhb, Nr0b2, Nr1h3, Lta4h, Alox5ap, Alka1, Apof, Slc16a6, Slc27a1, Slc27a3, Stard4, Cxcl16, Olr1, Pcsk9, Stab2 |
| Northern Blot | [55] | Nos2 |
| qPCR | [127] | Il1B, Tnf |
| qPCR | [137] | Stat1 |
| Northern Blot | [147] | hsp70 |
| qPCR | [150] | Tnf |
| RNAseq | [156] | Gm42427, Wdfy1, Tmem181bp, Dynlt1b, Cd59a, Sel1l3, Tmem181bp, Gm42031, Gm43305, Xlr, Rab4a, Nbea, AC157515.1, Gm42742, C2, Cfap69, Steap2, Stc1, Vegfc, Steap1, Fgf23, Hist1h4n, Gm10359, Saa1, Slc6a14, 1700028P14P, Lhb, mt-Nd4l, Gm14176, Gm5776, Emx2 |
| qPCR | [158] | RXRa, Abca1, Abcg1 |
| qPCR | [167] | Cd11b, Cd206, TgfB |
| qPCR | [188] | Tlr1, Tlr2, Tlr3, Tlr4, Tlr5, Tlr6, Tlr7, Tlr8, Tlr9 |
| qPCR | [189] | Mrc1 |
| qPCR | [190] | FoxO3, Cd206, TgfB, Fizz1 |
| Northern Blot | [194] | IaB, IeB |
| RNAseq | [206] | Wdfy1, Marco, Csf2, Dynlt1b, Smo, Efcab7, Ptch1 |
| qPCR | [216] | Reverba |
| RNAseq | [253] | Gm10260, Gm10036, Ly6e |
| qPCR | [254] | Rac1 |
| qPCR | [260] | Arg1, Mrc1 |
| Microarray | [261] | miR-181c, miR-3068, miR-330, miR-5135, miR-10a, miR720, miR-29c |
| qPCR | [274] | miR-142-3p |
| qPCR | [290] | Abca1, Abcg1, Tnf, Il1B, Ptgs2, Ccl2, Mmp9, TgfB |
| qPCR | [291] | Tnf, Il6, Il1B |
| qPCR | [301] | miR146b, miR22, miR423, miR15a |
| Microarray | [302] | miR720 |
| qPCR | [310] | miR350-3p |
| qPCR | [329] | Tnf |
| RNAseq | [335] | Egfem1, Ovol2, Adora3, Ikzf4, Inka1, Mterf2, Nr1d1, Zfp979, Penk, Itga6, A430106G13Rik, Btla, Esco2, Cenpk, Fam221a, Zbtb32, Clec4a1, Cenpw, Cbfa2t3, Ska1, Mir142b, Haao, Id3, E2f7, Cenpm, Rmi2, Fcho1, Ica1, Fam78b, Cdca7, Cd93, Fignl1, Cbx2, Nid2, Ptp4a3, Pclaf, Ccna2, Depdc1a, Myrip, Lrrc75a, Abi3, Fam83d, Fam92a, Uhrf1, C1qtnf12, Rad54b, Chek1, Sapcd2, Psat1, Deldc1b Ifg1, Pdgfa, C3, Egfr, Rab27B, Cd36, Rab25, P2ry1, Sod3, Ccnt1, Csf1, Siglec1, Rab12, Hsf4 |

**Supplementary Table 4 – Input table for protein upregulated MAIC analysis**

| **Method** | **Publication** | **Protein names** |
| --- | --- | --- |
| Flow Cytometry | [9] | FIZZ1 |
| ELISA | [11] | IL1B, IL6, TNF, IL10 |
| ELISA | [15] | TNF, IL1 |
| FACS | [32] | iNOS, CD206 |
| Mass Spec | [41] | PRRG2, FBN1, COL1A1, POSTN, PAI1, COL1A2 |
| Assay | [61] | PGE2, COX |
| Assay | [71] | PGE2, COX |
| Assay | [96] | IA |
| Flow Cytometry | [110a] | CCR7, CD163 |
| Assay | [110b] | ARG1 |
| ELISA | [110c] | IL1B, IL6, IL10 |
| Flow Cytometry | [127] | CD11b, CD11c, CD206, CD64, MHCIIA |
| Flow Cytometry | [129a] | CD14, TLR4 |
| ELISA | [129b] | TNF, IL10 |
| Assay | [129c] | ARG1, Inos |
| Assay | [133] | NFKB, p65, PGE2, COX2 |
| ELISA | [149] | IL6 |
| Western Blot | [150] | CD14 |
| ELISA | [156] | CXCL2, CXCL1, IL1RA, IL10, CCL3, TIMP1, TNF, CXCL10, CCL4, CCL5, IL6, CCL2, CXCL13, MCSF, IFNY, CCL1, GCSF, CXCL11, IL17, CXCL12, TREM1, IL1B, C5a, IL1a, IL13, GMCSF, IL16, IL7, IL4, CXCL9, IL3, IL5 |
| Proteomic | [183] | MYO1F, RARS, DPYS11, PIK3AP1, HUWE1, TPP1, NAPRT, PPAT, SF3B2, MARS, PGAM2, ACTG1, F1NC, CAPG, TUBA1A, ACTA1, YWHAZ, CAP1, ANXA5, F1NA, CTSD, H2-D1, HSC70, TIP-B1, FGH, GM2A, GPX1, ANXA6, CTSB, PRX, MDH2, ANXA2, UBA1, PGK, LCP1, ANXA1, HSP90AB1, ALDH-2, GRP78, HA1, LYZ, MOM2, VAT-1, RP14 |
| Immuno Cytochemistry | [186] | iNOS |
| Flow Cytometry | [189a] | IFNyR1, CD11b, CD45 |
| ELISA | [189b] | TNF |
| Flow Cytometry | [197] | TNF, IL10, CCL2 |
| ELISA | [198a] | TNF |
| Assay | [198b] | PGI2 |
| ELISA | [206] | CXCL13, C5a, G-CSF, GMCSF, CCL1, IFNy, IL1a, IL1B, IL3, IL4, IL7, IL13, IL18, IL17, CXCL10, CXCL11, CXCL9, CCL3, CXCL2, CXCL12, TIMP1, TREM1, CCL4, CD54, CCL5, TNF, IL6, IL1ra, MCSF, CCL4, IL23, IL27 |
| Assay | [211a] | iNOS, ARG1 |
| ELISA | [211b] | IL1a, IL10, TNF |
| Assay | [212] | TNF |
| ELISA | [226] | IL6 |
| Flow Cytometry | [230a] | Marco |
| Immuno staining | [230b] | BGalactosidase |
| Assay | [245] | TNF, IL1, IL6 |
| ELISA | [249] | IL10 |
| Flow Cytometry | [254] | Marco |
| Western Blot | [260a] | p62 |
| ELISA | [260b] | TNF, IL1B, IL6 |
| Immuno flourescence | [260c] | iNOS |
| ELISA | [274] | IL6 |
| ELISA | [277] | PGE2 |
| Western Blot | [280] | A20 |
| Western Blot | [281] | COX2, PGE2 |
| Immuno flourescence | [306] | Fcy1R, FcyAR, IA, MAC1 |
| Flow Cytometry | [327] | IL10, CD40, CX3CR1, TGFB |
| Immuno flourescence | [333] | C3b, IgG2a, IgG2b, IgG3, IgM, IgA, IgE |
| ELISA | [338a] | IL10 |
| Flow Cytometry | [338b] | MAC1, B7.2, TLR4 |
| Assay | [339a] | IL6 |
| Flow Cytometry | [339b] | TLR2, TLR4 |

**Supplementary Table 5 – Input table for proteins downregulated MAIC analysis**

| **Method** | **Publication** | **Protein names** |
| --- | --- | --- |
| Western Blot | [1] | FOXO3 |
| Flow Cytometry | [9] | MHCII, KI67 |
| Flow Cytometry | [11] | CD11b |
| ELISA | [15a] | TNF, IL1, IL12 |
| ELISA | [15b] | IL12 |
| ELISA | [15c] | TNF, IL1, IL12 |
| ELISA | [32] | IL1RA, TNF |
| Mass Spec | [41a] | QPCT, Granulin, ATP6AP1, LAMP1, PPT1, IL-41, CTSZ, GGH, CTSL1, CCR9, C1QA, TCN2, C1QB, LRP1, B2M, CREG1, CSF1R, CTSB, H2KW28, C1QC, SDF4, STAB1, SGP1A, PAFAH, HEXA, CST3, LGALS3BP |
| ELISA | [41b] | LRP1 |
| Assay | [85] | TNF |
| Assay | [96] | TNF, ASGM1 |
| ELISA | [99] | IL1, TNF |
| ELISA | [110] | TGFB, IL10, IL6, IL1B |
| Flow Cytometry | [129a] | CD14 |
| ELISA | [129b] | TNF, IL10 |
| Assay | [129c] | ARG1, iNOS |
| Western Blot | [137] | STAT1B, STAT1A |
| ELISA | [149] | TNF |
| Western Blot | [150] | TNF, RACK1 |
| Proteomic | [183] | ACTC1, CCT3, MELA, HSP70-1B, ARG1, DESMOYOKIN, GSTP1, H2BA, EUK, SET, SLC25A5, PSMD3, UBE2Q1, KRT28, EEF2 |
| Flow Cytometry | [188a] | TLR4 |
| ELISA | [188b] | TNF, IL6 |
| Western Blot | [190] | FOXO3 |
| Flow Cytometry | [194a] | IA |
| Western Blot | [194b] | IA |
| ELISA | [196a] | TNF, IL6, P38, JNK |
| Western Blot | [196b] | P38, JNK |
| Flow Cytometry | [197] | TNF, IFNy, IL12, IL6, CCL2 |
| ELISA | [201] | TNF, IL1B, IL6, IL12 |
| Western Blot | [206] | WDFY1 |
| Assay | [211a] | iNOS, ARG1 |
| ELISA | [211b] | IL10, TNF |
| Flow Cytometry | [230] | TLR4 |
| Assay | [233] | ARG1 |
| ELISA | [245a] | TNF, IL1, IL6 |
| Assay | [245b] | TNF, IL1, IL6 |
| ELISA | [249a] | TNF, IL6 |
| Western Blot | [249b] | p38 |
| Western Blot | [254] | RAC1 |
| Western Blot | [260a] | ATG5, LC3-II |
| Immuno flourescence | [260b] | CD206 |
| Immuno flourescence | [264] | TNF |
| ELISA | [268] | IL1B, IL6, TNF |
| Western Blot | [280] | CYLD |
| Western Blot | [290] | ABCA1, ABCG1 |
| Immuno flourescence | [306] | FcuR, Fcy2AR, Fcy2BR, FceR |
| Flow Cytometry | [327] | IFNy |
| Immuno flourescence | [333] | C5b, C3bi, IgG1 |
| Flow Cytometry | [334a] | ED2 |
| ELISA | [334b] | TNF |
| ELISA | [338a] | IL1B, IL6, IL12, TNF |
| Flow Cytometry | [338b] | CD14 |
| Assay | [339] | TNF, IFNy |

**Supplementary Table 6 – MAIC publications**

| **Publication number** | **Citation** |
| --- | --- |
| 1 | (Becker et al., 2018) |
| 2 | (Yutian Chen et al., 2021) |
| 3 | (Reidy et al., 2019) |
| 4 | (Daniel Clark et al., 2020) |
| 6 | (Ben Menachem-Zidon et al., 2020) |
| 9 | (Z. Zhang et al., 2020) |
| 11 | (R. Kumar et al., 2020) |
| 13 | (Hachim et al., 2017) |
| 15 | (Kohut et al., 2004) |
| 20 | (Ma et al., 2015) |
| 24 | (Fei et al., 2016) |
| 26 | (Uchida et al., 2019) |
| 29 | (Lagadari et al., 2004) |
| 32 | (Gibon et al., 2016) |
| 33 | (J. B. Lin, Sene, et al., 2018) |
| 40 | (Sloboda et al., 2018) |
| 41 | (Vi et al., 2018) |
| 42 | (Pinto et al., 2014) |
| 43 | (Tobin et al., 2021) |
| 47 | (Dimitrijević, Aleksić, et al., 2014) |
| 48 | (Hinks & Franklin, 2000) |
| 51 | (Kaneko et al., 2018) |
| 55 | (Kissin et al., 1997) |
| 56 | (Fix et al., 2021) |
| 58 | (Baek et al., 2020) |
| 60 | (Kelly et al., 2007) |
| 61 | (Mura et al., 1998) |
| 64 | (LoPresti & Brown, 2018) |
| 67 | (Canan et al., 2014) |
| 68 | (Schneider et al., 2007) |
| 69 | (C. Q. Wang et al., 1995) |
| 71 | (Beharka et al., 2002) |
| 76 | (De La Fuente et al., 2001) |
| 84 | (Leone et al., 2007) |
| 85 | (Davila et al., 1990) |
| 89 | (Kizaki et al., 2000) |
| 94 | (De Wazieres et al., 1998) |
| 95 | (Terao et al., 2002) |
| 96 | (Higashimoto et al., 1993) |
| 99 | (Wallace et al., 1995) |
| 100 | (Liscovsky et al., 2011) |
| 110 | (Dimitrijević et al., 2016) |
| 113 | (Alibhai et al., 2020) |
| 116 | (Meschiari et al., 2018) |
| 117 | (Wu et al., 2013) |
| 122 | (Q. Zhang et al., 2000) |
| 125 | (Inomata et al., 2020) |
| 127 | (Lafuse et al., 2019) |
| 129 | (Dimitrijević, Stanojević, et al., 2014) |
| 131 | (Malinina et al., 2020) |
| 132 | (Murciano et al., 2008) |
| 133 | (Dayong et al., 2003) |
| 134 | (Claycombe et al., 2002) |
| 137 | (Yoon et al., 2004) |
| 140 | (De Toda et al., 2019) |
| 141 | (Vineeta et al., 1999) |
| 142 | (Y. He et al., 2019) |
| 146 | (Nakashima et al., 2019) |
| 147 | (Moore et al., 1998) |
| 148 | (Gon et al., 1996) |
| 149 | (Gómez et al., 2006) |
| 150 | (Marinovich et al., 1999) |
| 151 | (Breuillard et al., 2017) |
| 155 | (Kang et al., 2021) |
| 156 | (Babagana et al., 2021) |
| 157 | (Hall et al., 2020) |
| 158 | (H. Chen et al., 2013) |
| 159 | (Farinas et al., 2018) |
| 160 | (Vida et al., 2017) |
| 166 | (Duarte et al., 2021) |
| 167 | (Yokozeki et al., 2021) |
| 175 | (Throsby et al., 1993) |
| 178 | (Kang et al., 2021) |
| 179 | (Stewart et al., 2021) |
| 182 | (Lavin et al., 2020) |
| 183 | (Smallwood et al., 2011) |
| 185 | (Chabrier et al., n.d.) |
| 186 | (L.-C. Chen et al., 1996) |
| 187 | (Mahbub et al., 2012) |
| 188 | (Renshaw et al., 2002) |
| 189 | (Barrett et al., 2015) |
| 190 | (Becker et al., 2018) |
| 191 | (Couchie et al., 2017) |
| 192 | (Sheng et al., 2018) |
| 193 | (Arranz et al., 2010) |
| 194 | (Herrero et al., 2001) |
| 196 | (Boehmer et al., 2004) |
| 197 | (Stout et al., 2005) |
| 198 | (Tang et al., 2000) |
| 199 | (Boyd et al., 2012) |
| 200 | (Dayan et al., 2000) |
| 201 | (Kovacs et al., 2010) |
| 202 | (Spencer & Daynes, 1997) |
| 204 | (Daniel Clark et al., 2020) |
| 206 | (Babagana et al., 2021) |
| 207 | (Hall et al., 2020) |
| 211 | (Cecílio et al., 2011) |
| 212 | (Han et al., 1995) |
| 215 | (Drynda et al., 2017) |
| 216 | (Sato et al., 2014) |
| 219 | (Y. Zhang et al., 2021) |
| 222 | (Son et al., 2017) |
| 225 | (Horrillo et al., 2011) |
| 226 | (Thevaranjan et al., 2017) |
| 228 | (H. Zhao et al., 2013) |
| 230 | (Yamada et al., 2019) |
| 231 | (Stout-Delgado et al., 2016) |
| 233 | (Müller et al., 2008) |
| 237 | (Lively & Schlichter, 2012) |
| 238 | (C. Zhang et al., 2020) |
| 239 | (Yang et al., 2008) |
| 241 | (W. He et al., 2018) |
| 242 | (Zhong et al., 2020) |
| 244 | (Y. B. Shaik-Dasthagirisaheb et al., 2015) |
| 245 | (Yifang Chen & Bradley, 1993) |
| 246 | (Blacher et al., 2022) |
| 247 | (Wong et al., 2017) |
| 249 | (Boehmer et al., 2005) |
| 250 | (Ying Wang et al., 2019) |
| 251 | (Fontana et al., 2013) |
| 253 | (C. M. C. Li et al., 2020) |
| 254 | (Z. Li et al., 2017) |
| 256 | (Verschoor et al., 2014) |
| 258 | (Takahashi, Totsuka, et al., 2016) |
| 260 | (R. Liu et al., 2021) |
| 261 | (J. Li et al., 2020) |
| 264 | (Agius et al., 2009) |
| 265 | (Huang et al., 2021) |
| 267 | (C. Zhao et al., 2006) |
| 268 | (Lee et al., 2020) |
| 269 | (Rojas et al., 2016) |
| 270 | (Gonzalez et al., 2015) |
| 272 | (Campuzano et al., 2008) |
| 273 | (Suzuki et al., 2008) |
| 274 | (Y. Liu et al., 2016) |
| 277 | (D. Liu et al., 2015) |
| 278 | (Jämsen et al., 2020) |
| 280 | (Hinojosa et al., 2014) |
| 281 | (Seo et al., 2003) |
| 282 | (Grufman et al., 2014) |
| 283 | (Cribbs et al., 2012) |
| 284 | (Doddapattar et al., 2018) |
| 285 | (L. Lin et al., 2016) |
| 286 | (Yazdani B. Shaik-Dasthagirisaheb et al., 2010) |
| 290 | (Sene et al., 2013) |
| 291 | (O. H. Kim et al., 2017) |
| 292 | (Corsini et al., 2002) |
| 293 | (Patsalos et al., 2018) |
| 296 | (Yuan Wang et al., 2020) |
| 297 | (Wung Chung et al., 2017) |
| 301 | (Santeford et al., 2021) |
| 302 | (J. B. Lin, Moolani, et al., 2018) |
| 304 | (Stratton et al., 2020) |
| 306 | (Vtetvicka et al., 1987) |
| 308 | (Sierra et al., 2007) |
| 309 | (Porrini et al., 2017) |
| 310 | (Chang et al., 2018) |
| 311 | (Chiao et al., 2011) |
| 312 | (Nguyen et al., 2016) |
| 314 | (Cho et al., 2018) |
| 316 | (Casaletto et al., 2018) |
| 318 | (O’Donnell et al., 2019) |
| 321 | (Nwadiugwu, 2022) |
| 322 | (Zandi et al., 2015) |
| 324 | (Takahashi, Ishigami, et al., 2016) |
| 325 | (Hanouna et al., 2017) |
| 327 | (Jackaman, Radley‐Crabb, et al., 2013) |
| 328 | (Sebastián et al., 2009) |
| 329 | (Costello et al., 2016) |
| 333 | (Vttvicka et al., 1985) |
| 334 | (Dimitrijević et al., 2013) |
| 335 | (McQuattie-Pimentel et al., 2021) |
| 337 | (Brandenberger et al., 2018) |
| 338 | (Chelvarajan et al., 2005) |
| 339 | (Pattabiraman et al., 2016) |
| 341 | (Baror et al., 2019) |
| 342 | (A. Kumar et al., 2013) |
| 344 | (Stanojević et al., 2015) |

**Supplementary Table 7 – Changes in macrophage numbers as a result of increasing age**

| **Macrophage subtype** | **Direction of change with age** | **Additional factors** | **Citation** |
| --- | --- | --- | --- |
| F4/80+ CD11b+ | Decrease  Decrease  Increase  Increase | Peritoneum  Liver  Liver  Aorta and mesentery | (Vivian et al., 2023)  (Collins et al., 2013)  (Stahl et al., 2020)  (Trott et al., 2018) |
| F4/80+CD11b+ CD64+ | Decrease | Skin | (Dube et al., 2022) |
| Iba1+ | Increase  Increase  No change | Osseous spiral lamina  Retina  Apical turn | (Seicol et al., 2022)  (Kezic et al., 2020)  (Noble et al., 2019) |
| Peritoneal | Decrease  No change | Percentage live  Control | (Blacher et al., 2022)  (D. Clark et al., 2021) |
| CD68+/DAPI+  CD206+/CD68+/DAPI+  CD206−/CD68+/DAPI+ | No change  No change  Decrease | Skeletal muscle | (Ahmadi et al., 2022) |
| CD11b+F4/80+Ly6Gneg | Increase | Spleen | (Duong et al., 2022) |
| CD68+ | Increase  Increase  Increase  Increase  Decrease  No change  No change | Prostate  Vaginal wound  Liver  Kidneys  Prostate  Muscle  Peritoneum | (Pascal et al., 2022)  (Ben Menachem-Zidon et al., 2020)  (Maeso-Díaz et al., 2019)  (Wung Chung et al., 2017)  (Werneck-Gomes et al., 2020)  (Sloboda et al., 2018)  (Dimitrijević et al., 2016) |
| F4/80+ | Increase  Decrease  Decrease  Decrease  Decrease  Decrease  Decrease  Decrease  No change  No change  No change | Ovarian stroma  Peritoneum  White adipose tissue  RM-9 tumour  Glomeruli  Bone marrow-derived  Peritoneum  Peritoneum  Fracture callus  Carotid artery  Spleen | (Umehara et al., 2022)  (Y. Zhang et al., 2021)  (Abdullahi et al., 2021)  (Bellinger et al., 2021)  (Kaneko et al., 2018)  (Hachim et al., 2017)  (Pattabiraman et al., 2016)  (Murciano et al., 2008)  (Daniel Clark et al., 2020)  (Walker et al., 2019)  (Kovacs et al., 2010) |
| Arg1Hi | Increase | Skin wounds | (Vu et al., 2022) |
| Monocyte-derived | Increase | Spinal cord injury | (Stewart et al., 2021) |
| Cardiac resident | No change | Steady-state control | (Esfahani et al., 2021) |
| CD11b+ F4/80+ Ly‐6C+ | Increase | Muscle | (Kawanishi & Machida, 2021) |
| M2 | Increase  Increase | Muscle injury  Spleen and bone marrow | (Tobin et al., 2021)  (Jackaman, Radley-Crabb, et al., 2013) |
| M1 (CD86+)  M2 (CD163+) | Increase  Decrease  Decrease | Liver  Stroma | (Kang et al., 2021)  (J. Li et al., 2020) |
| CD11b+ CD206+ | Decrease  Increase | Intervertebral disc  Peritoneum | (Yokozeki et al., 2021)  (Lee et al., 2020) |
| CD45+F4/80+  Ly6E+Ly6Chi | Decrease | Muscle | (C. Zhang et al., 2020) |
| HO-1+/iNOS+ and HO-1+/CD163+ | Increase | Peritoneum | (Bloomer et al., 2020) |
| CD45+Ly6G‐Siglec‐F‐NK1.1‐ CD11b+Ly6C‐CD64+ | Decrease | Skeletal muscle | (Runyan et al., 2020) |
| M2 (RELM-α+)  M1 (MHCII+) | Increase  Decrease | Ovary | (Z. Zhang et al., 2020) |
| CD68+CD163+DAPI+ | Decrease | Soleus | (Reidy et al., 2019) |
| CD11c+ CD11b+ | Increase | Alveolar | (Lafuse et al., 2019) |
| F4/80+ CD206+ | Decrease | Kidney injury | (M. G. Kim et al., 2019) |
| CD68+iNOS+  CD68+IL-10+ | Increase  Decrease | Myocardial infarction | (C. C. Lin et al., 2019) |
| NG2+ | Increase | Microglia | (Baror et al., 2019) |
| CD68+CD11b+ | Decrease | Skeletal muscle | (Reidy et al., 2018) |
| CD11b+ CD45high | Increase  Increase | Brain  Brain | (Wolfe et al., 2018)  (Barrett et al., 2015) |
| CD11b+MHCII+F4/80+ | Increase | Bone marrow-derived | (O. H. Kim et al., 2017) |
| F4/80+GHS-R+ | Increase | Peritoneum | (L. Lin et al., 2016) |
| F4/80+CD206−  F4/80+CD206+ | Increase  Decrease | Cardiac | (Ma et al., 2015) |
| ED2high | Decrease | Peritoneum | (Dimitrijević et al., 2013) |
| F4/80+IL4R+ | Decrease | Bone marrow-derived | (Mahbub et al., 2012) |
| CD11bhigh F4/80high | Decrease | Peritoneum | (Liscovsky et al., 2011) |
| CD26+ | Increase | Peritoneum | (Dimitrijević et al., 2008) |
| CD68+CD163+  CD11b+ | Decrease  Decrease | Skeletal muscle | (Przybyla et al., 2006) |

**Supplementary Table 8 – Findings from publications assessing changes in soluble mediator release with age**

| **Soluble mediator** | **Direction of change with age** | **Cell environment** | **Additional factors** | **Citation** |
| --- | --- | --- | --- | --- |
| TNF, IFN-γ, IL-12, and MCP-1 | Decrease | LPS-stimulated in vitro | Bone marrow or peritoneal | (Stout et al., 2005) |
| TNF, IL-6, IL-1β and IL-12 | Decrease | LPS-stimulated | Splenic | (Kovacs et al., 2010) |
| IL-1 | Decrease | LPS-stimulated | Splenic | (Bruley-Rosset et al., 1985) |
| TNF | Decrease | IFN-primed, LPS-stimulated | Alveolar | (Higashimoto et al., 1993) |
| TNF, IL-1, IL-6 | Decrease | LPS-stimulated | Peritoneal | (Yifang Chen & Bradley, 1993) |
| IL-1, TNF | Decrease | LPS+IFNy-stimulated | Peritoneal | (Wallace et al., 1995) |
| TNF | Increase | Basal | Peritoneal | (Han et al., 1995) |
| TNF | Decrease | LPS-stimulated | Alveolar | (Marinovich et al., 1999) |
| IL-1β | Increase | Control and Con A-stimulated | Peritoneal | (De La Fuente et al., 2001) |
| TNF, NO, IL-12, IL-1β | Increase | LPS and IFN-y stimulated | Alveolar | (Kohut et al., 2004) |
| IL-10 | Increase | LPS-stimulated | Splenic | (Chelvarajan et al., 2005) |
| TNF and IL-6 | Decrease | TLR 2 and 4 ligand stimulation | Splenic | (Boehmer et al., 2005) |
| TNF and IL-6 | Decrease | Basal | Peritoneal | (Vivian et al., 2023) |
| IL-6 and TNF | Increased | Steady state and after LPS stimulation | Renal | (Lefèvre et al., 2021) |
| IL-1β, IL-6, and TNF | Decreased | Salmonella extract and LPS-stimulated | Peritoneal | (Lee et al., 2020) |
| IL-6 | Increase | LPS or S. pneumoniae stimulated | BMDMs | (Thevaranjan et al., 2017) |
| TNF  Check IL-1ra | Increase | M1 (LPS-stimulated) | BMDMs | (Gibon et al., 2016) |
| TNF, IL-1B, IP-10 | Increase | Stimulated with soluble brain extract from aged mice | BMDMs | (Costello et al., 2016) |
| TNF | Increase | IFNγ-stimulated | BMDMs | (Barrett et al., 2015) |
| IL-6 and TNF | Decrease | LPS+IFNy-stimulated | Peritoneal and splenic | (Kaushal & Kansal, 2011) |
| IL-10  IL-12 | Increase  Decrease | CpG-ODN+IFNy | Peritoneal | (Liscovsky et al., 2011) |
| TNF and IL-6 | Decrease | Pg-challenged | BMDM | (Yazdani B. Shaik-Dasthagirisaheb et al., 2010) |
| TNF, IFN-γ, and IL-6 | Decrease | C albicans antigen-challenged | Dermal | (Agius et al., 2009) |
| TNF | Increase | Uf-Ni and Uf-Co stimulation | Alveolar | (Q. Zhang et al., 2000) |
| IL-2  IL-4 | Decrease  No change | Co-culture of macrophages and T cells | Peritoneal | (Beharka et al., 1997) |
| TNF | Decrease | LPS, poly(I:C), CpG ODN, S. aureus and zymosan A | Splenic | (Renshaw et al., 2002) |
| TNF and IL-6, IL-1B and IL-12 | Decrease | LPS-stimulated | Splenic | (Kovacs et al., 2010) |
| IL-1B, TNF, IL-6, IL-3, IFN-y | Increase | Basal | Peritoneal | (Arranz et al., 2010) |
| IL-6, TGFB  IL-1β, IL-4 | Decreased  No change | LPS-stimulated | Peritoneal | (Dimitrijević et al., 2016) |
| VEGF, IL-10  IL-6 | Increase  Decrease | sFasL and LPS stimulation | BMDMs | (H. Zhao et al., 2013) |
| IL-6  TNF  PGE2 | No change  Increase | Basal and LPS-stimulated | Peritoneal | (Wu et al., 2013) |
| PGE2 | Increase | Resting and LPS-stimulated | Peritoneal | (H. Chen et al., 2013) |
| TNF | Decrease | LPS | Peritoneal | (Dimitrijević et al., 2013) |
| TNF, IL-6, IL-1β  MIP-2  IL-10 | Decrease  No change | Zymosan, yeasts cells, and hyphae  C. albicans and hyphae | Peritoneal | (Murciano et al., 2008) |

| **Soluble mediator** | **Direction of change with age** | **Cell environment** | **Additional factors** | **Citation** |
| --- | --- | --- | --- | --- |
| ROS | Decrease | Proliferating and senescent cell media | Peritoneal | (R. Kumar et al., 2020) |
| ROS | Increase | Spinal cord injury lesion epicentre, vehicle treated | Microglia | (B. Zhang et al., 2019) |
| Nitric oxide | No change | IFNγ/LPS | BMDMs | (Hachim et al., 2017) |
| XO , ROS and O2 | Increase | Basal | Peritoneal | (Vida et al., 2017) |
| ROS | Increase | Spinal cord injury | Microglia | (B. Zhang et al., 2016) |
| mtROS | Increase | Control and bleomycin-treated | BMDMs | (Stout-Delgado et al., 2016) |
| NO | Increase  No change  Increase | Basal  LPS-stimulated  Basal  LPS-stimulated | DA rats, peritoneal  AO rats, peritoneal | (Dimitrijević, Stanojević, et al., 2014) |
| ROS | Decrease  Increase | Basal | Peritoneal  Splenic | (Kaushal & Kansal, 2014) |
| NO | No change  Decrease | Basal  LPS | Peritoneal and splenic | (Kaushal & Kansal, 2011) |
| NO | Increase | LPS | Peritoneal, first two hours of exposure | (Smallwood et al., 2011) |
| NO | Increase | CpG-ODN + IFN-γ | Peritoneal | (Liscovsky et al., 2011) |
| NO | Decrease | Pg 381 | BMM | (Yazdani B. Shaik-Dasthagirisaheb et al., 2010) |
| NO | No change | Classically activated | BMDM | (Müller et al., 2008) |
| NO | Increase  Decrease | LPS and IFN-γ | Alveolar  Peritoneal | (Kohut et al., 2004) |
| NO | Increase | LPS | Peritoneal | (Beharka et al., 2002) |
| O2 | No change | HMP-glycol stimulation | Peritoneal | (Saez et al., 2002) |
| FOR | Increase | Basal and stress | Peritoneal and alveolar | (De Wazieres et al., 2000) |
| NO | Decrease | Basal and exercise | Peritoneal | (Lu et al., 1999) |
| NO2 | Decrease | LPS | Splenic | (Kissin et al., 1997) |
| H2O2 | Decrease | Basal and PMA stimulation | Tumour-activated | (Rosa et al., 1993) |
| O – 2 anions | Increase | Zymosan | Peritoneal | (Lavie, 1992) |
| H2O2 | Increase | Basal | Peritoneal | (Gervais et al., 1988) |
| NO | Increase | IFN | Peritoneal | (Rollo & Denhardt, 1996) |
| NO | No change | Basal | Peritoneal | (Tang et al., 2000) |
| NO | Increase | IFN and LPS | Hepatic | (Stahl et al., 2020) |
| FOR | Increase | Basal | Peritoneal and alveolar | (De Wazieres et al., 2000) |

**Supplementary Table 9: Mean MAIC scores for all genes**

| **Gene** | **Downregulated** | **Upregulated** |
| --- | --- | --- |
| AA467197 | 0 | 1.003342 |
| Abca1 | -1.009128 | 0 |
| Abcb1b | 0 | 1.003342 |
| Abcg1 | -1.009128 | 0 |
| AC125149.1 | 0 | 1.067736 |
| AC133103.1 | 0 | 1.003342 |
| AC168977.1 | 0 | 1.003342 |
| AC174776.1 | 0 | 1.067736 |
| Adfp | 0 | 1.004692 |
| Aldh1a2 | 0 | 1.003342 |
| Alox15 | 0 | 1.004692 |
| Alppl2 | 0 | 1.003342 |
| Arg1 | -1.415746 | 1.379062 |
| Atf3 | 0 | 1.003342 |
| B630019K06Rik | 0 | 1.003342 |
| C6 | 0 | 1.153306 |
| Ccdc7a | 0 | 1.003342 |
| Ccl2 | -1.009128 | 2.471590 |
| Ccl5 | 0 | 1.012183 |
| Ccl8 | 0 | 1.153306 |
| Cd11b | -1.094324 | 1.379062 |
| Cd163 | 0 | 1.073837 |
| Cd206 | -1.415746 | 0 |
| Cd209d | 0 | 1.153306 |
| Cd209f | 0 | 1.153306 |
| Cd209g | 0 | 1.153306 |
| Cd274 | 0 | 1.012183 |
| Cd31 | 0 | 1.071658 |
| Cd36 | 0 | 1.071658 |
| Cd4 | 0 | 1.003342 |
| Cd40 | 0 | 1.505031 |
| Cd79a | 0 | 1.103789 |
| Cd79b | 0 | 1.103789 |
| Cdv3-ps | 0 | 1.067736 |
| CIITa | 0 | 1.318284 |
| Cldn3 | 0 | 1.003342 |
| Clec4d | 0 | 1.012183 |
| Col4a1 | 0 | 1.003342 |
| Col4a2 | 0 | 1.003342 |
| Coro1a | 0 | 1.012183 |
| Cox2 | 0 | 3.989184 |
| Csmd1 | 0 | 1.153306 |
| Csprs | 0 | 1.103789 |
| Ctsd | 0 | 1.003342 |
| Ctss | 0 | 1.003342 |
| Cxcl1 | 0 | 1.153306 |
| Cxcl13 | 0 | 2.257096 |
| Cxcl2 | 0 | 1.153306 |
| Cxcl3 | 0 | 1.003342 |
| Cxcl5 | 0 | 1.153306 |
| Cyp7b1 | 0 | 1.004692 |
| Dact2 | 0 | 1.003342 |
| Dnajb4 | 0 | 1.003342 |
| Dnajc10 | 0 | 1.003342 |
| Dpysl3 | 0 | 1.003342 |
| Egfl6 | 0 | 1.003342 |
| Enpp5 | 0 | 1.003342 |
| Esr1 | 0 | 1.003342 |
| Esrrg | 0 | 1.003342 |
| F4/80 | 0 | 1.071658 |
| Fabp4 | 0 | 1.004692 |
| Fads3 | 0 | 1.004692 |
| Fam20c | 0 | 1.003342 |
| Fcna | 0 | 1.153306 |
| Fizz1 | -1.094324 | 0 |
| FoxO3 | -1.080482 | 0 |
| Gdf15 | 0 | 1.012183 |
| Gimap4 | 0 | 1.103789 |
| Gm11189 | 0 | 1.067736 |
| Gm15446 | 0 | 1.067736 |
| Gm16548 | 0 | 1.153306 |
| Gm16867 | 0 | 1.067736 |
| Gm27177 | 0 | 1.067736 |
| Gm43802 | 0 | 1.067736 |
| Gm4951 | 0 | 1.003342 |
| Gm7609 | 0 | 1.067736 |
| Gpr176 | 0 | 1.153306 |
| Gpx3 | 0 | 1.003342 |
| H2-Eb1 | 0 | 1.012183 |
| H2-M2 | 0 | 1.003342 |
| H2-M9 | 0 | 1.153306 |
| Has1 | 0 | 1.003342 |
| Hc | 0 | 1.003342 |
| Hist1h4m | 0 | 1.067736 |
| Hmcn1 | 0 | 1.003342 |
| Hmgcs2 | 0 | 1.004692 |
| Hmox1 | 0 | 1.012183 |
| Hspa1B | 0 | 1.003342 |
| Hspb1 | 0 | 1.003342 |
| Ifi208 | 0 | 1.067736 |
| Ifitm2 | 0 | 1.003342 |
| Ifnb | 0 | 2.386020 |
| IfnG | 0 | 1.318284 |
| Igha | 0 | 1.003342 |
| Ighg2b | 0 | 1.067736 |
| Ighg2c | 0 | 1.067736 |
| Ighm | 0 | 1.003342 |
| Ighv1-18 | 0 | 1.067736 |
| Ighv1-53 | 0 | 1.003342 |
| Ighv1-55 | 0 | 1.003342 |
| Igkc | 0 | 1.067736 |
| Igkv10-96 | 0 | 1.067736 |
| Igkv12-46 | 0 | 1.067736 |
| Igkv3-2 | 0 | 1.003342 |
| Il10 | 0 | 1.318284 |
| Il12p40 | 0 | 1.318284 |
| Il1b | -1.009128 | 1.517531 |
| Il6 | -1.003042 | 2.835213 |
| Inhba | 0 | 1.153306 |
| Irf1 | 0 | 1.318284 |
| Itih4 | 0 | 1.003342 |
| Jchain | 0 | 1.003342 |
| Kcnj15 | 0 | 1.003342 |
| Lamp3 | 0 | 1.003342 |
| Lcn2 | 0 | 1.003342 |
| Lif | 0 | 1.153306 |
| Lilr4b | 0 | 1.012183 |
| Lpl | 0 | 1.004692 |
| Lrp2 | 0 | 1.003342 |
| Ly6a | 0 | 1.103789 |
| Ly6d | 0 | 1.103789 |
| Ly6i | 0 | 1.003342 |
| Map2 | 0 | 1.003342 |
| Marco | 0 | 1.153306 |
| Mcp1 | 0 | 1.517531 |
| Mcsf | 0 | 1.318284 |
| MHCII | 0 | 1.505031 |
| miR-101b | 0 | 1.531035 |
| miR-33 | 0 | 1.073837 |
| miR26b | 0 | 1.531035 |
| miR350-3p | 0 | 1.465001 |
| Mlc1 | 0 | 1.003342 |
| Mmp9 | -1.009128 | 0 |
| Mrc1 | -1.125250 | 1.058439 |
| Ms4a1 | 0 | 1.103789 |
| Mustn1 | 0 | 1.003342 |
| Mzb1 | 0 | 1.103789 |
| Nos2 | -3.202137 | 3.989184 |
| Oser1 | 0 | 1.012183 |
| p16 | 0 | 1.058439 |
| p21 | 0 | 1.058439 |
| p53 | 0 | 1.058439 |
| Palmd | 0 | 1.003342 |
| Pla2g2d | 0 | 1.003342 |
| Plac8 | 0 | 1.103789 |
| Plin4 | 0 | 1.003342 |
| Prkaa2 | 0 | 1.004692 |
| Ptgs2 | -1.009128 | 0 |
| Rasef | 0 | 1.003342 |
| RXRa | -1.003042 | 0 |
| Saa3 | 0 | 1.153306 |
| Sftpd | 0 | 1.003342 |
| Slc7a2 | -1.415746 | 0 |
| Srgap1 | 0 | 1.003342 |
| Stabilin2 | 0 | 1.071658 |
| Tacr3 | 0 | 1.153306 |
| Tbxas1 | 0 | 1.004692 |
| TgfB | -1.094324 | 1.286951 |
| TimD4 | 0 | 1.071658 |
| Tiparp | 0 | 1.012183 |
| Tlr1 | 0 | 1.318284 |
| Tmem132e | 0 | 1.153306 |
| Tnf | -1.009128 | 1.681907 |
| Tnfaip8 | 0 | 1.003342 |
| Vcam1 | 0 | 1.003342 |
| Vsig4 | 0 | 1.153306 |
| Zbtb7c | 0 | 1.003342 |
| Zmat4 | 0 | 1.003342 |

**Supplementary Table 10: Mean MAIC score for all proteins**

| **Protein** | **Downregulated** | **Upregulated** |
| --- | --- | --- |
| ACTC1 | -1.100502 | 0 |
| ARG1 | -2.762423 | 2.800089 |
| ASGM1 | -3.402046 | 0 |
| ATP6AP1 | -1.057452 | 0 |
| B2M | -1.057452 | 0 |
| B7.2 | 0 | 1.404013 |
| C1QA | -1.057452 | 0 |
| C1QB | -1.057452 | 0 |
| C1QC | -1.057452 | 0 |
| C5a | 0 | 1.389373 |
| CCL1 | 0 | 1.389373 |
| CCL2 | -3.041867 | 3.742451 |
| CCL3 | 0 | 1.389373 |
| CCL4 | 0 | 1.389373 |
| CCL5 | 0 | 1.389373 |
| CCR9 | -1.057452 | 0 |
| CCT3 | -1.100502 | 0 |
| CD11b | 0 | 1.412643 |
| CD11c | 0 | 1.412643 |
| CD14 | 0 | 3.436139 |
| CD206 | 0 | 4.347373 |
| CD40 | 0 | 1.540760 |
| CD45 | 0 | 1.094798 |
| CD54 | 0 | 1.323200 |
| CD64 | 0 | 1.412643 |
| COX | 0 | 2.172427 |
| COX2 | 0 | 4.387966 |
| CREG1 | -1.057452 | 0 |
| CSF1R | -1.057452 | 0 |
| CST3 | -1.057452 | 0 |
| CTSB | -1.057452 | 0 |
| CTSL1 | -1.057452 | 0 |
| CTSZ | -1.057452 | 0 |
| CX3CR1 | 0 | 1.540760 |
| CXCL1 | 0 | 1.389373 |
| CXCL10 | 0 | 1.389373 |
| CXCL11 | 0 | 1.389373 |
| CXCL12 | 0 | 1.389373 |
| CXCL13 | 0 | 1.389373 |
| CXCL2 | 0 | 1.389373 |
| CXCL9 | 0 | 1.389373 |
| DESMOYOKIN | -1.100502 | 0 |
| EEF2 | -1.100502 | 0 |
| EUK | -1.100502 | 0 |
| Fcy1R | 0 | 1.528066 |
| FcyAR | 0 | 1.528066 |
| GCSF | 0 | 1.389373 |
| GGH | -1.057452 | 0 |
| GMCSF | 0 | 1.389373 |
| Granulin | -1.057452 | 0 |
| GSTP1 | -1.100502 | 0 |
| H2BA | -1.100502 | 0 |
| H2KW28 | -1.057452 | 0 |
| HEXA | -1.057452 | 0 |
| HSP70-1B | -1.100502 | 0 |
| IA | -3.997293 | 3.360359 |
| IFNy | -6.676268 | 1.389373 |
| IFNyR1 | 0 | 1.094798 |
| IL1 | -7.200325 | 4.966004 |
| IL10 | -3.402046 | 4.890307 |
| IL12 | -6.373862 | 0 |
| IL13 | 0 | 1.389373 |
| IL16 | 0 | 1.389373 |
| IL17 | 0 | 1.389373 |
| IL18 | 0 | 1.323200 |
| IL1a | 0 | 2.244680 |
| IL1B | -3.343294 | 2.225058 |
| IL1Ra | -3.402046 | 1.389373 |
| IL23 | 0 | 1.323200 |
| IL27 | 0 | 1.323200 |
| IL3 | 0 | 1.389373 |
| IL4 | 0 | 1.389373 |
| IL41 | -1.057452 | 0 |
| IL5 | 0 | 1.389373 |
| IL6 | -10.457448 | 4.646402 |
| IL7 | 0 | 1.389373 |
| iNOS | -1.451405 | 12.914487 |
| JNK | -5.573576 | 0 |
| KRT28 | -1.100502 | 0 |
| LAMP1 | -1.057452 | 0 |
| LGALS3BP | -1.057452 | 0 |
| LRP1 | -2.700771 | 0 |
| MAC1 | 0 | 2.932079 |
| MCSF | 0 | 1.389373 |
| MELA | -1.100502 | 0 |
| MHCIIA | 0 | 1.412643 |
| NFKB | 0 | 1.974389 |
| P38 | -5.573576 | 0 |
| p65 | 0 | 1.974389 |
| PAFAH | -1.057452 | 0 |
| PGE2 | 0 | 7.282315 |
| PPT1 | -1.057452 | 0 |
| PSMD3 | -1.100502 | 0 |
| QPCT | -1.057452 | 0 |
| RACK1 | -3.402046 | 0 |
| SDF4 | -1.057452 | 0 |
| SET | -1.100502 | 0 |
| SGP1A | -1.057452 | 0 |
| SLC25A5 | -1.100502 | 0 |
| STAB1 | -1.057452 | 0 |
| TCN2 | -1.057452 | 0 |
| TGFB | -2.143216 | 1.540760 |
| TIMP1 | 0 | 1.389373 |
| TLR2 | 0 | 1.173465 |
| TLR4 | 0 | 1.583411 |
| TNF | -19.776293 | 8.007603 |
| TREM1 | 0 | 1.389373 |
| UBE2Q1 | -1.100502 | 0 |

**Supplementary references**

Abdullahi, A., Knuth, C. M., Auger, C., Sivayoganathan, T., Parousis, A., & Jeschke, M. G. (2021). Adipose browning response to burn trauma is impaired with aging. *JCI Insight*, *6*(16). https://doi.org/10.1172/jci

Agius, E., Lacy, K. E., Vukmanovic-Stejic, M., Jagger, A. L., Papageorgiou, A. P., Hall, S., Reed, J. R., Curnow, S. J., Fuentes-Duculan, J., Buckley, C. D., Salmon, M., Taams, L. S., Krueger, J., Greenwood, J., Klein, N., Rustin, M. H. A., & Akbar, A. N. (2009). Decreased TNF-α synthesis by macrophages restricts cutaneous immunosurveillance by memory CD4+ T cells during aging. *Journal of Experimental Medicine*, *206*(9), 1929–1940. https://doi.org/10.1084/jem.20090896

Ahmadi, M., Karlsen, A., Mehling, J., Soendenbroe, C., Mackey, A. L., & Hyldahl, R. D. (2022). Aging is associated with an altered macrophage response during human skeletal muscle regeneration. *Experimental Gerontology*, *169*, 111974. https://doi.org/10.1016/j.exger.2022.111974

Alibhai, F. J., Lim, F., Yeganeh, A., DiStefano, P. V., Binesh-Marvasti, T., Belfiore, A., Wlodarek, L., Gustafson, D., Millar, S., Li, S. H., Weisel, R. D., Fish, J. E., & Li, R. K. (2020). Cellular senescence contributes to age-dependent changes in circulating extracellular vesicle cargo and function. *Aging Cell*, *19*(3). https://doi.org/10.1111/acel.13103

Arranz, L., Lord, J. M., & De La Fuente, M. (2010). Preserved ex vivo inflammatory status and cytokine responses in naturally long-lived mice. *Age*, *32*(4), 451–466. https://doi.org/10.1007/s11357-010-9151-y

Babagana, M., Oh, K.-S., Chakraborty, S., Pacholewska, A., Aqdas, M., & Sung, M.-H. (2021). Hedgehog dysregulation contributes to tissue-specific inflammaging of resident macrophages. *Aging*, *13*(15), 19207–19229.

Baek, K. W., Lee, D. I., Jeong, M. J., Kang, S. A., Choe, Y., Yoo, J. Il, Yu, H. S., & Kim, J. S. (2020). Effects of lifelong spontaneous exercise on the M1/M2 macrophage polarization ratio and gene expression in adipose tissue of super-aged mice. *Experimental Gerontology*, *141*. https://doi.org/10.1016/j.exger.2020.111091

Baror, R., Neumann, B., Segel, M., Chalut, K. J., Fancy, S. P. J., Schafer, D. P., & Franklin, R. J. M. (2019). Transforming growth factor-beta renders ageing microglia inhibitory to oligodendrocyte generation by CNS progenitors. *GLIA*, *67*(7), 1374–1384. https://doi.org/10.1002/glia.23612

Barrett, J. P., Costello, D. A., O’Sullivan, J., Cowley, T. R., & Lynch, M. A. (2015). Bone marrow-derived macrophages from aged rats are more responsive to inflammatory stimuli. *Journal of Neuroinflammation*, *12*(67). https://doi.org/10.1186/s12974-015-0287-7

Becker, L., Nguyen, L., Gill, J., Kulkarni, S., Pasricha, P. J., & Habtezion, A. (2018). Age-dependent shift in macrophage polarisation causes inflammation-mediated degeneration of enteric nervous system. *Gut*, *67*(5), 827–836. https://doi.org/10.1136/gutjnl-2016-312940

Beharka, A. A., Wu, D., Han, S. N., & Meydani, S. N. (1997). Macrophage prostaglandin production contributes to the age-associated decrease in T cell function which is reversed by the dietary antioxidant vitamin E. *Mechanisms of Ageing and Development*, *93*, 59–77.

Beharka, A. A., Wu, D., Serafini, M., & Meydani, S. N. (2002). MECHANISM OF VITAMIN E INHIBITION OF CYCLOOXYGENASE ACTIVITY IN MACROPHAGES FROM OLD MICE: ROLE OF PEROXYNITRITE. *Free Radical Biology and Medicine*, *32*(6), 503–511.

Bellinger, D. L., Dulcich, M. S., Molinaro, C., Gifford, P., Lorton, D., Gridley, D. S., & Hartman, R. E. (2021). Psychosocial Stress and Age Influence Depression and Anxiety-Related Behavior, Drive Tumor Inflammatory Cytokines and Accelerate Prostate Cancer Growth in Mice. *Frontiers in Oncology*, *11*. https://doi.org/10.3389/fonc.2021.703848

Ben Menachem-Zidon, O., Parkes, I., Chill, H. H., Reubinoff, B., Sandberg, K., Ji, H., & Shveiky, D. (2020). Age-associated differences in macrophage response in a vaginal wound healing rat model. *International Urogynecology Journal*, *31*, 1803–1809. https://doi.org/10.1007/s00192-020-04266-9/Published

Blacher, E., Tsai, C., Litichevskiy, L., Shipony, Z., Iweka, C. A., Schneider, K. M., Chuluun, B., Heller, H. C., Menon, V., Thaiss, C. A., & Andreasson, K. I. (2022). Aging disrupts circadian gene regulation and function in macrophages. *Nature Immunology*, *23*(2), 229–236. https://doi.org/10.1038/s41590-021-01083-0

Bloomer, S. A., Moyer, E. D., Brown, K. E., & Kregel, K. C. (2020). Aging results in accumulation of M1 and M2 hepatic macrophages and a differential response to gadolinium chloride. *Histochemistry and Cell Biology*, *153*(1), 37–48. https://doi.org/10.1007/s00418-019-01827-y

Boehmer, E. D., Goral, J., Faunce, D. E., & Kovacs, E. J. (2004). Age-dependent decrease in Toll-like receptor 4-mediated proinflammatory cytokine production and mitogen-activated protein kinase expression. *Journal of Leukocyte Biology*, *75*(2), 342–349. https://doi.org/10.1189/jlb.0803389

Boehmer, E. D., Meehan, M. J., Cutro, B. T., & Kovacs, E. J. (2005). Aging negatively skews macrophage TLR2- and TLR4-mediated pro-inflammatory responses without affecting the IL-2-stimulated pathway. *Mechanisms of Ageing and Development*, *126*(12), 1305–1313. https://doi.org/10.1016/j.mad.2005.07.009

Boyd, A. R., Shivshankar, P., Jiang, S., Berton, M. T., & Orihuela, C. J. (2012). Age-related defects in TLR2 signaling diminish the cytokine response by alveolar macrophages during murine pneumococcal pneumonia. *Experimental Gerontology*, *47*(7), 507–518. https://doi.org/10.1016/j.exger.2012.04.004

Brandenberger, C., Kling, K. M., Vital, M., & Mühlfeld, C. (2018). The role of pulmonary and systemic immunosenescence in acute lung injury. *Aging and Disease*, *9*(4), 553–565. https://doi.org/10.14336/AD.2017.0902

Breuillard, C., Curis, E., Le Plénier, S., Cynober, L., & Moinard, C. (2017). Nitric oxide production by peritoneal macrophages from aged rats: A short term and direct modulation by citrulline. *Biochimie*, *133*, 66–73. https://doi.org/10.1016/j.biochi.2016.10.020

Bruley-Rosset, M., Dardenne, M., & Schuurs, A. (1985). Functional and quantitative changes of immune cells of ageing NZB mice treated with nandrolone decanoate. I. Effect on survival and autoantibody development. *Clinical and Experimental Immunology*, *62*(3), 630–638.

Campuzano, O., Castillo-Ruiz, M. M., Acarin, L., Castellano, B., & Gonzalez, B. (2008). Distinct pattern of microglial response, cyclooxygenase-2, and inducible nitric oxide synthase expression in the aged rat brain after excitotoxic damage. *Journal of Neuroscience Research*, *86*(14), 3170–3183. https://doi.org/10.1002/jnr.21751

Canan, C. H., Gokhale, N. S., Carruthers, B., Lafuse, W. P., Schlesinger, L. S., Torrelles, J. B., & Turner, J. (2014). Characterization of lung inflammation and its impact on macrophage function in aging. *Journal of Leukocyte Biology*, *96*(3), 473–480. https://doi.org/10.1189/jlb.4a0214-093rr

Casaletto, K. B., Staffaroni, A. M., Elahi, F., Fox, E., Crittenden, P. A., You, M., Neuhaus, J., Glymour, M., Bettcher, B. M., Yaffe, K., & Kramer, J. H. (2018). Perceived Stress is Associated with Accelerated Monocyte/Macrophage Aging Trajectories in Clinically Normal Adults. *American Journal of Geriatric Psychiatry*, *26*(9), 952–963. https://doi.org/10.1016/j.jagp.2018.05.004

Cecílio, C. A., Costa, E. H., Simioni, P. U., Gabriel, D. L., & Tamashiro, W. M. S. C. (2011). Aging alters the production of iNOS, arginase and cytokines in murine macrophages. *Brazilian Journal of Medical and Biological Research*, *44*(7), 671–681. https://doi.org/10.1590/s0100-879x2011007500067

Chabrier, G., Hobson, S., Yuldasheva, N., Kearney, M. T., Schurmans, S., Pineda-Torra, I., & Gage, M. C. (n.d.). Aged insulin resistant macrophages reveal dysregulated cholesterol biosynthesis, a pro-inflammatory profile and reduced foam cell formation capacity. *BioRxiv Preprint*. https://doi.org/10.1101/467118

Chang, H., Wang, X., & Yang, S. (2018). miR-350-3p Contributes to Age-Associated Impairment of IL-6 Production by Macrophages. *Immunological Investigations*, *47*(8), 790–800. https://doi.org/10.1080/08820139.2018.1508227

Chelvarajan, R. L., Collins, S. M., Van Willigen, J. M., & Bondada, S. (2005). The unresponsiveness of aged mice to polysaccharide antigens is a result of a defect in macrophage function. *Journal of Leukocyte Biology*, *77*(4), 503–512. https://doi.org/10.1189/jlb.0804449

Chen, H., Ma, F., Hu, X., Jin, T., Xiong, C., & Teng, X. (2013). Elevated COX2 expression and PGE2 production by downregulation of RXRα in senescent macrophages. *Biochemical and Biophysical Research Communications*, *440*(1), 157–162. https://doi.org/10.1016/j.bbrc.2013.09.047

Chen, L.-C., Pace, J. L., Russell, S. W., & Morrison, D. C. (1996). Altered Regulation of Inducible Nitric Oxide Synthase Expression in Macrophages from Senescent Mice. *Infection and Immunity*, *64*(10), 4288–4298.

Chen, Yifang, & Bradley, S. F. (1993). Aging and eliciting agents: Effect on murine peritoneal macrophage monokine bioactivity. *Experimental Gerontology*, *28*, 145–159.

Chen, Yutian, Pu, Q., Ma, Y., Zhang, H., Ye, T., Zhao, C., Huang, X., Ren, Y., Qiao, L., Liu, H. M., Esmon, C. T., Ding, B. Sen, & Cao, Z. (2021). Aging Reprograms the Hematopoietic-Vascular Niche to Impede Regeneration and Promote Fibrosis. *Cell Metabolism*, *33*(2), 395-410.e4. https://doi.org/10.1016/j.cmet.2020.11.019

Chiao, Y. A., Dai, Q., Zhang, J., Lin, J., Lopez, E. F., Ahuja, S. S., Chou, Y. M., Lindsey, M. L., & Jin, Y. F. (2011). Multi-analyte profiling reveals matrix metalloproteinase-9 and monocyte chemotactic protein-1 as plasma biomarkers of cardiac aging. *Circulation: Cardiovascular Genetics*, *4*(4), 455–462. https://doi.org/10.1161/CIRCGENETICS.111.959981

Cho, S. J., Rooney, K., Choi, A. M. K., & Stout-Delgado, H. W. (2018). NLRP3 inflammasome activation in aged macrophages is diminished during Streptococcus pneumoniae infection. *American Journal of Physiology - Lung Cellular and Molecular Physiology*, *314*(3), L372–L387. https://doi.org/10.1152/ajplung.00393.2017

Clark, D., Halpern, B., Miclau, T., Nakamura, M., Kapila, Y., & Marcucio, R. (2021). The Contribution of Macrophages in Old Mice to Periodontal Disease. *Journal of Dental Research*, *100*(12), 1397–1404. https://doi.org/10.1177/00220345211009463

Clark, Daniel, Brazina, S., Yang, F., Hu, D., Hsieh, C. L., Niemi, E. C., Miclau, T., Nakamura, M. C., & Marcucio, R. (2020). Age-related changes to macrophages are detrimental to fracture healing in mice. *Aging Cell*, *19*(3). https://doi.org/10.1111/acel.13112

Claycombe, K. J., Wu, D., Nikolova-Karakashian, M., Palmer, H., Beharka, A., Eric Paulson, K., & Meydani, S. N. (2002). Ceramide mediates age-associated increase in macrophage cyclooxygenase-2 expression. *Journal of Biological Chemistry*, *277*(34), 30784–30791. https://doi.org/10.1074/jbc.M204463200

Collins, B. H., Holzknecht, Z. E., Lynn, K. A., Sempowski, G. D., Smith, C. C., Liu, S., Parker, W., & Rockey, D. C. (2013). Association of age-dependent liver injury and fibrosis with immune cell populations. *Liver International*, *33*(8), 1175–1186. https://doi.org/10.1111/liv.12202

Corsini, E., Lucchi, L., Meroni, M., Racchi, M., Solerte, B., Fioravanti, M., Viviani, B., Marinovich, M., Govoni, S., & Galli, C. L. (2002). In Vivo Dehydroepiandrosterone Restores Age-Associated Defects in the Protein Kinase C Signal Transduction Pathway and Related Functional Responses. *The Journal of Immunology*, *168*(4), 1753–1758. https://doi.org/10.4049/jimmunol.168.4.1753

Costello, D. A., Keenan, K., McManus, R. M., Falvey, A., & Lynch, M. A. (2016). The age-related neuroinflammatory environment promotes macrophage activation, which negatively impacts synaptic function. *Neurobiology of Aging*, *43*, 140–148. https://doi.org/10.1016/j.neurobiolaging.2016.04.001

Couchie, D., Vaisman, B., Abderrazak, A., Mahmood, D. F. D., Hamza, M. M., Canesi, F., Diderot, V., El Hadri, K., Nègre-Salvayre, A., Page, A. Le, Fulop, T., Remaley, A. T., & Rouis, M. (2017). Human Plasma Thioredoxin-80 Increases With Age and in ApoE-/-Mice Induces Inflammation, Angiogenesis, and Atherosclerosis. *Circulation*, *136*(5), 464–475. https://doi.org/10.1161/CIRCULATIONAHA.117.027612

Cribbs, D. H., Berchtold, N. C., Perreau, V., Coleman, P. D., Rogers, J., Tenner, A. J., & Cotman, C. W. (2012). Extensive innate immune gene activation accompanies brain aging, increasing vulnerability to cognitive decline and neurodegeneration: A microarray study. *Journal of Neuroinflammation*, *9*. https://doi.org/10.1186/1742-2094-9-179

Davila, D. R., Edwards, C. K., Arkins, S., Simon, J., & Kelley, K. W. (1990). Interferon-gamma-induced priming for secretion of superoxide anion and tumor necrosis factor-alpha declines in macrophages from aged rats. *FASEB J*, *4*(11), 2906–2911.

Dayan, M., Segal, R., Globerson, A., Habut, B., Shearer, G. M., & Mozes, E. (2000). Effect of Aging on Cytokine Production in Normal and Experimental Systemic Lupus Erythematosus-Afflicted Mice. *Experimental Gerontology*, *35*, 225–236.

Dayong, W., Marko, M., Claycombe, K., Paulson, K. E., & Meydani, S. N. (2003). Ceramide-induced and age-associated increase in macrophage COX-2 expression is mediated through up-regulation of NF-κB activity. *Journal of Biological Chemistry*, *278*(13), 10983–10992. https://doi.org/10.1074/jbc.M207470200

De La Fuente, M., Rıo, M. Del, & Mediná, S. (2001). Changes with aging in the modulation by neuropeptide Y of murine peritoneal macrophage functions. In *Journal of Neuroimmunology* (Vol. 116). www.elsevier.comrlocaterjneuroin

De Toda, I. M., Vida, C., Miguel, L. S. S., & De La Fuente, M. (2019). Function, oxidative, and inflammatory stress parameters in immune cells as predictive markers of lifespan throughout aging. *Oxidative Medicine and Cellular Longevity*, *2019*. https://doi.org/10.1155/2019/4574276

De Wazieres, B., Harraga, S., Spehner, V., Bloy, C., Dupond, J. L., Vuitton, D. A., & Seilles, E. (2000). Effect of an auditory stress on peritoneal and alveolar cells in C57 BL/6J mice of advanced age. *Luminescence*, *15*, 233–237.

De Wazieres, B., Spehner, V., Harraga, S., Laplante, F., Corallo, F., Bloy, C., Dupond, J. L., Vuitton, D. A., & Seilles, E. (1998). Alteration in the production of free oxygen radicals and proinflammatory cytokines by peritoneal and alveolar macrophages in old mice and immunomodulatory effect of RU 41740 administration Part I: Effect of short and repetitive noise stress. *Immunopharmacology*, *39*, 51–59.

Dimitrijević, M., Aleksić, I., Vujić, V., Stanojević, S., Pilipović, I., von Hörsten, S., & Leposavić, G. (2014). Peritoneal exudate cells from long-lived rats exhibit increased IL-10/IL-1β expression ratio and preserved NO/urea ratio following LPS-stimulation in vitro. *Age*, *36*(4). https://doi.org/10.1007/s11357-014-9696-2

Dimitrijević, M., Stanojević, S., Blagojević, V., Ćuruvija, I., Vujnović, I., Petrović, R., Arsenović-Ranin, N., Vujić, V., & Leposavić, G. (2016). Aging affects the responsiveness of rat peritoneal macrophages to GM-CSF and IL-4. *Biogerontology*, *17*(2), 359–371. https://doi.org/10.1007/s10522-015-9620-x

Dimitrijević, M., Stanojević, S., Kuštrimović, N., Mitić, K., Vujić, V., Aleksić, I., Radojević, K., & Leposavić, G. (2013). The influence of aging and estradiol to progesterone ratio on rat macrophage phenotypic profile and NO and TNF-α production. *Experimental Gerontology*, *48*(11), 1243–1254. https://doi.org/10.1016/j.exger.2013.07.001

Dimitrijević, M., Stanojević, S., Mitić, K., Kuštrimović, N., Vujić, V., Miletić, T., & Kovačević-Jovanović, V. (2008). The anti-inflammatory effect of neuropeptide Y (NPY) in rats is dependent on dipeptidyl peptidase 4 (DP4) activity and age. *Peptides*, *29*(12), 2179–2187. https://doi.org/10.1016/j.peptides.2008.08.017

Dimitrijević, M., Stanojević, S., Vujić, V., Aleksić, I., Pilipović, I., & Leposavić, G. (2014). Aging oppositely affects TNF-α and IL-10 production by macrophages from different rat strains. *Biogerontology*, *15*(5), 475–486. https://doi.org/10.1007/s10522-014-9513-4

Doddapattar, P., Jain, M., Dhanesha, N., Lentz, S. R., & Chauhan, A. K. (2018). Fibronectin containing extra domain a induces plaque destabilization in the innominate artery of aged apolipoprotein E-deficient mice. *Arteriosclerosis, Thrombosis, and Vascular Biology*, *38*(3), 500–508. https://doi.org/10.1161/ATVBAHA.117.310345

Drynda, A., Obmińska-Mrukowicz, B., Zaczyńska, E., Zimecki, M., Kochanowska, I., Ryng, S., & Mączyński, M. (2017). 5-Amino-3-methyl-4-isoxazolecarboxylic acid hydrazide derivatives with in vitro immunomodulatory activities. *Chemical Biology and Drug Design*, *89*(5), 705–713. https://doi.org/10.1111/cbdd.12892

Duarte, C., Akkaoui, J., Ho, A., Garcia, C., Yamada, C., & Movila, A. (2021). Age-dependent effects of the recombinant spike protein/SARS-CoV-2 on the M–CSF– and IL-34-differentiated macrophages in vitro. *Biochemical and Biophysical Research Communications*, *546*, 97–102. https://doi.org/10.1016/j.bbrc.2021.01.104

Dube, C. T., Ong, Y. H. B., Wemyss, K., Krishnan, S., Tan, T. J., Janela, B., Grainger, J. R., Ronshaugen, M., Mace, K. A., & Lim, C. Y. (2022). Age-Related Alterations in Macrophage Distribution and Function Are Associated With Delayed Cutaneous Wound Healing. *Frontiers in Immunology*, *13*, 943159. https://doi.org/10.3389/fimmu.2022.943159

Duong, L., Pixley, F. J., Nelson, D. J., & Jackaman, C. (2022). Aging Leads to Increased Monocytes and Macrophages With Altered CSF-1 Receptor Expression and Earlier Tumor-Associated Macrophage Expansion in Murine Mesothelioma. *Frontiers in Aging*, *3*, 848925. https://doi.org/10.3389/fragi.2022.848925

Esfahani, N. S., Wu, Q., Kumar, N., Ganesan, L. P., Lafuse, W. P., & Rajaram, M. V. S. (2021). Aging influences the cardiac macrophage phenotype and function during steady state and during inflammation. *Aging Cell*, *20*(8). https://doi.org/10.1111/acel.13438

Farinas, A. F., Bamba, R., Pollins, A. C., Cardwell, N. L., Nanney, L. B., & Thayer, W. P. (2018). Burn wounds in the young versus the aged patient display differential immunological responses. *Burns*, *44*(6), 1475–1481. https://doi.org/10.1016/j.burns.2018.05.012

Fei, F., Lee, K. M., McCarry, B. E., & Bowdish, D. M. E. (2016). Age-associated metabolic dysregulation in bone marrow-derived macrophages stimulated with lipopolysaccharide. *Scientific Reports*, *6*. https://doi.org/10.1038/srep22637

Fix, D. K., Ekiz, H. A., Petrocelli, J. J., Mckenzie, A. M., Mahmassani, Z. S., O’Connell, R. M., & Drummond, M. J. (2021). Disrupted macrophage metabolic reprogramming in aged soleus muscle during early recovery following disuse atrophy. *Aging Cell*, *20*(9). https://doi.org/10.1111/acel.13448

Fontana, L., Zhao, E., Amir, M., Dong, H., Tanaka, K., & Czaja, M. J. (2013). Aging promotes the development of diet-induced murine steatohepatitis but not steatosis. *Hepatology*, *57*(3), 995–1004. https://doi.org/10.1002/hep.26099

Gervais, F., Patel, P., & Skamene, E. (1988). Increased natural resistance to Listeria monocytogenes in senescent mice correlates with enhanced macrophage bactericidal activity. *Journal of Gerontology*, *43*(6), B152-6. https://doi.org/10.1093/geronj/43.6.b152

Gibon, E., Loi, F., Córdova, L. A., Pajarinen, J., Lin, T., Lu, L., Nabeshima, A., Yao, Z., & Goodman, S. B. (2016). Aging Affects Bone Marrow Macrophage Polarization: Relevance to Bone Healing. *Regenerative Engineering and Translational Medicine*, *2*(2), 98–104. https://doi.org/10.1007/s40883-016-0016-5

Gómez, C. R., Acuña-Castillo, C., Nishimura, S., Pérez, V., Escobar, A., Salazar-Onfray, F., Sabaj, V., Torres, C., Walter, R., & Sierra, F. (2006). Serum from aged F344 rats conditions the activation of young macrophages. *Mechanisms of Ageing and Development*, *127*(3), 257–263. https://doi.org/10.1016/j.mad.2005.10.002

Gon, Y., Hashimoto, S., Hayashi, S., Koura, T., Matsumoto, K., & Horie, T. (1996). Lower serum concentrations of cytokines in elderly patients with pneumonia and the impaired production of cytokines by peripheral blood monocytes in the elderly. *Clinical and Experimental Immunology*, *106*(1), 120–126.

Gonzalez, O. A., Novak, M. J., Kirakodu, S., Stromberg, A., Nagarajan, R., Huang, C. B., Chen, K. C., Orraca, L., Martinez-Gonzalez, J., & Ebersole, J. L. (2015). Differential Gene Expression Profiles Reflecting Macrophage Polarization in Aging and Periodontitis Gingival Tissues. *Immunological Investigations*, *44*(7), 643–664. https://doi.org/10.3109/08820139.2015.1070269

Grufman, H., Schiopu, A., Edsfeldt, A., Björkbacka, H., Nitulescu, M., Nilsson, M., Persson, A., Nilsson, J., & Gonçalves, I. (2014). Evidence for altered inflammatory and repair responses in symptomatic carotid plaques from elderly patients. *Atherosclerosis*, *237*(1), 177–182. https://doi.org/10.1016/j.atherosclerosis.2014.08.042

Hachim, D., Wang, N., Lopresti, S. T., Stahl, E. C., Umeda, Y. U., Rege, R. D., Carey, S. T., Mani, D., & Brown, B. N. (2017). Effects of aging upon the host response to implants. *Journal of Biomedical Materials Research - Part A*, *105*(5), 1281–1292. https://doi.org/10.1002/jbm.a.36013

Hall, B. M., Gleiberman, A. S., Strom, E., Krasnov, P. A., Frescas, D., Vujcic, S., Leontieva, O. V., Antoch, M. P., Kogan, V., Koman, I. E., Zhu, Y., Tchkonia, T., Kirkland, J. L., Chernova, O. B., & Gudkov, A. V. (2020). Immune checkpoint protein VSIG4 as a biomarker of aging in murine adipose tissue. *Aging Cell*, *19*(10). https://doi.org/10.1111/acel.13219

Han, D., Hosokawa, T., Aoike, A., & Kawai, K. (1995). Age-related enhancement of tumor necrosis factor (TNF) production in mice. *Mechanisms of Ageing and Development*, *84*, 39–54.

Hanouna, G., Mesnard, L., Vandermeersch, S., Perez, J., Placier, S., Haymann, J. P., Campagne, F., Moroch, J., Bataille, A., Baud, L., & Letavernier, E. (2017). Specific calpain inhibition protects kidney against inflammaging. *Scientific Reports*, *7*(1). https://doi.org/10.1038/s41598-017-07922-1

He, W., Yuan, T., Choezom, D., Hunkler, H., Annamalai, K., Lupse, B., & Maedler, K. (2018). Ageing potentiates diet-induced glucose intolerance, β-cell failure and tissue inflammation through TLR4. *Scientific Reports*, *8*(1). https://doi.org/10.1038/s41598-018-20909-w

He, Y., Munday, J. S., Perrott, M., Wang, G., & Liu, X. (2019). Association of age with the expression of hypoxia-inducible factors HIF-1α, HIF-2α, HIF-3α and VEGF in lung and heart of Tibetan sheep. *Animals*, *9*(9). https://doi.org/10.3390/ani9090673

Herrero, C., Marques, L., Lloberas, J., & Celada, A. (2001). IFN gamma-dependent transcription of MHC class II IA is impaired in macrophages from aged mice. *Journal of Clinical Investigation*, *107*, 485–493.

Higashimoto, Y., Fukuchi, Y., Shimada, Y., Ishida, K., Ohata, M., Furuse, T., Shu, C., Teramoto, S., Matsuse, T., Sudo, E., Orimo, H., & Higashimoto, Y. (1993). THE EFFECTS OF AGING ON THE FUNCTION OF ALVEOLAR MACROPHAGES IN MICE. In *Mechanisms of Ageing and Development* (Vol. 69).

Hinks, G. L., & Franklin, R. J. M. (2000). Delayed changes in growth factor gene expression during slow remyelination in the CNS of aged rats. *Molecular and Cellular Neuroscience*, *16*(5), 542–556. https://doi.org/10.1006/mcne.2000.0897

Hinojosa, C. A., Akula Suresh Babu, R., Rahman, M. M., Fernandes, G., Boyd, A. R., & Orihuela, C. J. (2014). Elevated A20 contributes to age-dependent macrophage dysfunction in the lungs. *Experimental Gerontology*, *54*, 58–66. https://doi.org/10.1016/j.exger.2014.01.007

Horrillo, D., Sierra, J., Arribas, C., García-San Frutos, M., Carrascosa, J. M., Lauzurica, N., Fernández-Agulló, T., & Ros, M. (2011). Age-associated development of inflammation in Wistar rats: Effects of caloric restriction. *Archives of Physiology and Biochemistry*, *117*(3), 140–150. https://doi.org/10.3109/13813455.2011.577435

Huang, J., Ladeiras, D., Yu, Y., Ming, X. F., & Yang, Z. (2021). Detrimental Effects of Chronic L-Arginine Rich Food on Aging Kidney. *Frontiers in Pharmacology*, *11*. https://doi.org/10.3389/fphar.2020.582155

Inomata, M., Xu, S., Chandra, P., Meydani, S. N., Takemura, G., Philips, J. A., & Leong, J. M. (2020). Macrophage LC3-associated phagocytosis is an immune defense against Streptococcus pneumoniae that diminishes with host aging. *PNAS*, *117*(52), 33561–33569. https://doi.org/10.1073/pnas.2015368117/-/DCSupplemental

Jackaman, C., Radley-Crabb, H. G., Soffe, Z., Shavlakadze, T., Grounds, M. D., & Nelson, D. J. (2013). Targeting macrophages rescues age-related immune deficiencies in C57BL/6J geriatric mice. *Aging Cell*, *12*(3), 345–357. https://doi.org/10.1111/acel.12062

Jackaman, C., Radley‐Crabb, H. G., Soffe, Z., Shavlakadze, T., Grounds, M. D., & Nelson, D. J. (2013). Targeting macrophages rescues age‐related immune deficiencies in C57 <scp>BL</scp> /6J geriatric mice. *Aging Cell*, *12*(3), 345–357. https://doi.org/10.1111/acel.12062

Jämsen, E., Pajarinen, J., Lin, T. hua, Lo, C. W., Nabeshima, A., Lu, L., Nathan, K., Eklund, K. K., Yao, Z., & Goodman, S. B. (2020). Effect of Aging on the Macrophage Response to Titanium Particles. *Journal of Orthopaedic Research*, *38*(2), 405–416. https://doi.org/10.1002/jor.24461

Kaneko, Y., Cho, T., Sato, Y., Goto, K., Yamamoto, S., Goto, S., Madaio, M. P., & Narita, I. (2018). Attenuated macrophage infiltration in glomeruli of aged miceresultinginamelioratedkidneyinjuryinnephrotoxic serum nephritis. *Journals of Gerontology - Series A Biological Sciences and Medical Sciences*, *73*(9), 1178–1186. https://doi.org/10.1093/gerona/gly019

Kang, K., Xia, A., Meng, F., Chunyu, J., Sun, X., Ren, G., Yu, D., Jiang, X., Tang, L., Xiao, W., & Li, D. (2021). FGF21 alleviates chronic inflammatory injury in the aging process through modulating polarization of macrophages. *International Immunopharmacology*, *96*. https://doi.org/10.1016/j.intimp.2021.107634

Kaushal, D., & Kansal, V. K. (2011). Age-related decline in macrophage and lymphocyte functions in mice and its alleviation by treatment with probiotic Dahi containing Lactobacillus acidophilus and Bifidobacterium bifidum. *Journal of Dairy Research*, *78*(4), 404–411. https://doi.org/10.1017/S0022029911000537

Kaushal, D., & Kansal, V. K. (2014). Dahi containing lactobacillus acidophilus and Bifidobacterium bifidum improves phagocytic potential of macrophages in aged mice. *Journal of Food Science and Technology*, *51*(6), 1147–1153. https://doi.org/10.1007/s13197-012-0637-8

Kawanishi, N., & Machida, S. (2021). Alterations of macrophage and neutrophil content in skeletal muscle of aged versus young mice. *Muscle and Nerve*, *63*(4), 600–607. https://doi.org/10.1002/mus.27158

Kelly, J., Khan, A. A., Yin, J., Ferguson, T. A., & Apte, R. S. (2007). Senescence regulates macrophage activation and angiogenic fate at sites of tissue injury in mice. *Journal of Clinical Investigation*, *117*(11), 3421–3426. https://doi.org/10.1172/JCI32430

Kezic, J. M., Chrysostomou, V., McMenamin, P. G., & Crowston, J. G. (2020). Effects of age on retinal macrophage responses to acute elevation of intraocular pressure. *Experimental Eye Research*, *193*. https://doi.org/10.1016/j.exer.2020.107995

Kim, M. G., Yang, J., Ko, Y. S., Lee, H. Y., Oh, S. W., Cho, W. Y., & Jo, S. K. (2019). Impact of aging on transition of acute kidney injury to chronic kidney disease. *Scientific Reports*, *9*(1). https://doi.org/10.1038/s41598-019-54585-1

Kim, O. H., Kim, H., Kang, J., Yang, D., Kang, Y. H., Lee, D. H., Cheon, G. J., Park, S. C., & Oh, B. C. (2017). Impaired phagocytosis of apoptotic cells causes accumulation of bone marrow-derived macrophages in aged mice. *BMB Reports*, *50*(1), 43–48. https://doi.org/10.5483/BMBRep.2017.50.1.167

Kissin, E., Tomasi, M., McCartney-Francis, N., Gibbs, C. L., & Smith, P. D. (1997). Age-related decline in murine macrophage production of nitric oxide. *The Journal of Infectious Diseases*, *175*, 1004–1007.

Kizaki, T., Ookawara, T., Haga, S., Matsuura, N., Ohno, H., & Kizaki, T. (2000). Effects of Ageing on Generation of ED2 high Major Histocompatibility Complex Class II þ Macrophages During Cold Stress. *Scand J. Immunol.*, *51*, 36–44.

Kohut, M. L., Senchina, D. S., Madden, K. S., Martin, A. E., Felten, D. L., & Moynihan, J. A. (2004). Age effects on macrophage function vary by tissue site, nature of stimulant, and exercise behavior. *Experimental Gerontology*, *39*(9), 1347–1360. https://doi.org/10.1016/j.exger.2004.07.001

Kovacs, E. J., Gomez, C. R., Karavitis, J., Palmer, J. L., Faunce, D. E., Ramirez, L., & Nomellini, V. (2010). Interleukin-6 contributes to age-related alteration of cytokine production by macrophages. *Mediators of Inflammation*. https://doi.org/10.1155/2010/475139

Kumar, A., Stoica, B. A., Sabirzhanov, B., Burns, M. P., Faden, A. I., & Loane, D. J. (2013). Traumatic brain injury in aged animals increases lesion size and chronically alters microglial/macrophage classical and alternative activation states. *Neurobiology of Aging*, *34*(5), 1397–1411. https://doi.org/10.1016/j.neurobiolaging.2012.11.013

Kumar, R., Sharma, A., Padwad, Y., & Sharma, R. (2020). Preadipocyte secretory factors differentially modulate murine macrophage functions during aging which are reversed by the application of phytochemical EGCG. *Biogerontology*, *21*(3), 325–343. https://doi.org/10.1007/s10522-020-09861-3

Lafuse, W. P., Rajaram, M. V. S., Wu, Q., Moliva, J. I., Torrelles, J. B., Turner, J., & Schlesinger, L. S. (2019). Identification of an Increased Alveolar Macrophage Subpopulation in Old Mice That Displays Unique Inflammatory Characteristics and Is Permissive to Mycobacterium tuberculosis Infection. *The Journal of Immunology*, *203*(8), 2252–2264. https://doi.org/10.4049/jimmunol.1900495

Lagadari, M., Blois, S., Margni, R., & Miranda, S. (2004). Analysis of Macrophage Presence in Murine Placenta: Influence of Age and Parity Status. *American Journal of Reproductive Immunology*, *51*, 49–55.

Lavie, L. (1992). Age-related alterations in respiratory burst activation induced by various stimuli in mouse peritoneal macrophages treated with thermal stress. *Arch. Gerontol. Geriatr. Suppl*, *3*, 203–218.

Lavin, K. M., Perkins, R. K., Jemiolo, B., Raue, U., Trappe, S. W., & Trappe, T. A. (2020). Effects of aging and lifelong aerobic exercise on basal and exercise-induced inflammation. *Journal of Applied Physiology (Bethesda, Md. : 1985)*, *128*(1), 87–99. https://doi.org/10.1152/japplphysiol.00495.2019

Lee, D. Y., Lim, J. S., & Cho, K. A. (2020). Differential Activation of Macrophages Based on Their Environment in Advanced Age. *Chonnam Medical Journal*, *56*(1), 12. https://doi.org/10.4068/cmj.2020.56.1.12

Lefèvre, L., Iacovoni, J. S., Martini, H., Bellière, J., Maggiorani, D., Dutaur, M., Marsal, D. J., Decaunes, P., Pizzinat, N., Mialet-Perez, J., Cussac, D., Parini, A., & Douin-Echinard, V. (2021). Kidney inflammaging is promoted by CCR2+ macrophages and tissue-derived micro-environmental factors. *Cellular and Molecular Life Sciences*, *78*(7), 3485–3501. https://doi.org/10.1007/s00018-020-03719-0

Leone, M., Bechah, Y., Meghari, S., Lepidi, H., Capo, C., Raoult, D., & Mege, J. L. (2007). Coxiella burnetii infection in C57BL/6 mice aged 1 or 14 months. *FEMS Immunology and Medical Microbiology*, *50*(3), 396–400. https://doi.org/10.1111/j.1574-695X.2007.00272.x

Li, C. M. C., Shapiro, H., Tsiobikas, C., Selfors, L. M., Chen, H., Rosenbluth, J., Moore, K., Gupta, K. P., Gray, G. K., Oren, Y., Steinbaugh, M. J., Guerriero, J. L., Pinello, L., Regev, A., & Brugge, J. S. (2020). Aging-Associated Alterations in Mammary Epithelia and Stroma Revealed by Single-Cell RNA Sequencing. *Cell Reports*, *33*(13). https://doi.org/10.1016/j.celrep.2020.108566

Li, J., Yin, X., Zhang, B., Li, C., & Lu, P. (2020). Bioinformatical Analysis of miRNA-mRNA Interaction Network Underlying Macrophage Aging and Cholesterol-Responsive Difference between Young and Aged Macrophages. *BioMed Research International*, *2020*. https://doi.org/10.1155/2020/9267475

Li, Z., Jiao, Y., Fan, E. K., Scott, M. J., Li, Y., Li, S., Billiar, T. R., Wilson, M. A., Shi, X., & Fan, J. (2017). Aging-Impaired Filamentous Actin Polymerization Signaling Reduces Alveolar Macrophage Phagocytosis of Bacteria. *The Journal of Immunology*, *199*(9), 3176–3186. https://doi.org/10.4049/jimmunol.1700140

Lin, C. C., Chen, S. yi, Lien, H. Y., Lin, S. Z., & Lee, T. M. (2019). Targeting the PI3K/STAT3 axis modulates age-related differences in macrophage phenotype in rats with myocardial infarction. *Journal of Cellular and Molecular Medicine*, *23*(9), 6378–6392. https://doi.org/10.1111/jcmm.14526

Lin, J. B., Moolani, H. V., Sene, A., Sidhu, R., Kell, P., Lin, J. B., Dong, Z., Ban, N., Ory, D. S., & Apte, R. S. (2018). Macrophage microRNA-150 promotes pathological angiogenesis as seen in age-related macular degeneration. *JCI Insight*, *3*(7). https://doi.org/10.1172/jci.insight.120157

Lin, J. B., Sene, A., Santeford, A., Fujiwara, H., Sidhu, R., Ligon, M. M., Shankar, V. A., Ban, N., Mysorekar, I. U., Ory, D. S., & Apte, R. S. (2018). Oxysterol Signatures Distinguish Age-Related Macular Degeneration from Physiologic Aging. *EBioMedicine*, *32*, 9–20. https://doi.org/10.1016/j.ebiom.2018.05.035

Lin, L., Lee, J., Buras, E., Yu, K., Wang, R., Smith, C., Wu, H., Sheikh-Hamad, D., & Sun, Y. (2016). Ghrelin receptor regulates adipose tissue inflammation in aging. *Aging*, *8*(1), 178–191.

Liscovsky, M. V., Ranocchia, R. P., Alignani, D. O., Gorlino, C. V., Morón, G., Maletto, B. A., & Pistoresi-Palencia, M. C. (2011). CpG-ODN+IFN-γ confer pro- and anti-inflammatory properties to peritoneal macrophages in aged mice. *Experimental Gerontology*, *46*(6), 462–467. https://doi.org/10.1016/j.exger.2011.01.006

Liu, D., Wang, D., Xu, Z., Gao, J., Liu, M., Liu, Y., Jiang, M., & Zheng, D. (2015). Dysregulated expression of miR-101b and miR-26b lead to age-associated increase in LPS-induced COX-2 expression in murine macrophage. *Age*, *37*(5). https://doi.org/10.1007/s11357-015-9836-3

Liu, R., Cui, J., Sun, Y., Xu, W., Wang, Z., Wu, M., Dong, H., Yang, C., Hong, S., Yin, S., & Wang, H. (2021). Autophagy deficiency promotes M1 macrophage polarization to exacerbate acute liver injury via ATG5 repression during aging. *Cell Death Discovery*, *7*(1). https://doi.org/10.1038/s41420-021-00797-2

Liu, Y., Song, X., Meng, S., & Jiang, M. (2016). Downregulated expression of miR-142-3p in macrophages contributes to increased IL-6 levels in aged mice. *Molecular Immunology*, *80*, 11–16. https://doi.org/10.1016/j.molimm.2016.10.009

Lively, S., & Schlichter, L. C. (2012). Age-Related Comparisons of Evolution of the Inflammatory Response After Intracerebral Hemorrhage in Rats. *Translational Stroke Research*, *3*(SUPPL. 1), 132–146. https://doi.org/10.1007/s12975-012-0151-3

LoPresti, S. T., & Brown, B. N. (2018). Effect of source animal age upon macrophage response to extracellular matrix biomaterials. *Journal of Immunology and Regenerative Medicine*, *1*, 57–66. https://doi.org/10.1016/j.regen.2018.03.004

Lu, Q., Ceddia, M. A., Price, E. A., Ye, S.-M., & Woods, J. A. (1999). Chronic exercise increases macrophage-mediated tumor cytolysis in young and old mice. *Am. J. Physiol.*, *276*(45), 482–489.

Ma, Y., Chiao, Y. A., Clark, R., Flynn, E. R., Yabluchanskiy, A., Ghasemi, O., Zouein, F., Lindsey, M. L., & Jin, Y. F. (2015). Deriving a cardiac ageing signature to reveal MMP-9-dependent inflammatory signalling in senescence. *Cardiovascular Research*, *106*(3), 421–431. https://doi.org/10.1093/cvr/cvv128

Maeso-Díaz, R., Ortega-Ribera, M., Lafoz, E., JoséLozano, J., Baiges, A., Francés, R., Albillos, A., Peralta, C., García-Pagán, J. C., Bosch, J., Cogger, V. C., & Gracia-Sancho, J. (2019). Aging influences hepatic microvascular biology and liver fibrosis in advanced chronic liver disease. *Aging and Disease*, *10*(4), 684–698. https://doi.org/10.14336/AD.2019.0127

Mahbub, S., Deburghgraeve, C. R., & Kovacs, E. J. (2012). Advanced age impairs macrophage polarization. *Journal of Interferon and Cytokine Research*, *32*(1), 18–26. https://doi.org/10.1089/jir.2011.0058

Malinina, A., Dikeman, D., Westbrook, R., Moats, M., Gidner, S., Poonyagariyagorn, H., Walston, J., & Neptune, E. R. (2020). IL10 deficiency promotes alveolar enlargement and lymphoid dysmorphogenesis in the aged murine lung. *Aging Cell*, *19*(4). https://doi.org/10.1111/acel.13130

Marinovich, G., Racchi, M., Govoni, S., Emanuela Corsini, C. L., Battaini, F., & Lucchi, L. (1999). A Defective Protein Kinase C Anchoring System Underlying Age-Associated Impairment in TNF-α Production in Rat Macrophages. *J Immunol References*, *163*, 3468–3473. http://www.jimmunol.org/content/163/6/http://www.jimmunol.org/content/163/6/3468.full#ref-list-1

McQuattie-Pimentel, A. C., Ren, Z., Joshi, N., Watanabe, S., Stoeger, T., Chi, M., Lu, Z., Sichizya, L., Aillon, R. P., Chen, C. I., Soberanes, S., Chen, Z., Reyfman, P. A., Walter, J. M., Anekalla, K. R., Davis, J. M., Helmin, K. A., Runyan, C. E., Abdala-Valencia, H., … Budinger, G. R. S. (2021). The lung microenvironment shapes a dysfunctional response of alveolar macrophages in aging. *Journal of Clinical Investigation*, *131*(4). https://doi.org/10.1172/JCI140299

Meschiari, C. A., Jung, M., Iyer, R. P., Yabluchanskiy, A., Toba, H., Garrett, M. R., & Lindsey, M. L. (2018). Macrophage overexpression of matrix metalloproteinase-9 in aged mice improves diastolic physiology and cardiac wound healing after myocardial infarction. *American Journal of Physiology - Heart and Circulatory Physiology*, *314*(2), H224–H235. https://doi.org/10.1152/ajpheart.00453.2017

Moore, S. A., Lopez, A., Richardson, A., & Pahlavani, M. A. (1998). Effect of age and dietary restriction on expression of heat shock protein 70 in rat alveolar macrophages. In *Mechanisms of Ageing and Development* (Vol. 104, Issue 182).

Müller, I., Hailu, A., Choi, B. S., Abebe, T., Fuentes, J. M., Munder, M., Modolell, M., & Kropf, P. (2008). Age-related alteration of arginase activity impacts on severity of leishmaniasis. *PLoS Neglected Tropical Diseases*, *2*(5). https://doi.org/10.1371/journal.pntd.0000235

Mura, C., Beharka, A. A., Nim Han, S., Eric Paulson, K., Hwang, D., & Nikbin Meydani, S. (1998). *Age-associated increase in PGE 2 synthesis and COX activity in murine macrophages is reversed by vitamin E*.

Murciano, C., Yáñez, A., O’Connor, J. E., Gozalbo, D., & Gil, M. L. (2008). Influence of aging on murine neutrophil and macrophage function against Candida albicans. *FEMS Immunology and Medical Microbiology*, *53*(2), 214–221. https://doi.org/10.1111/j.1574-695X.2008.00418.x

Nakashima, M., Kinoshita, M., Nakashima, H., Kotani, A., Ishikiriyama, T., Kato, S., Hiroi, S., & Seki, S. (2019). Pioglitazone improves phagocytic activity of liver recruited macrophages in elderly mice possibly by promoting glucose catabolism. *Innate Immunity*, *25*(6), 356–368. https://doi.org/10.1177/1753425919849620

Nguyen, T. V. V., Frye, J. B., Zbesko, J. C., Stepanovic, K., Hayes, M., Urzua, A., Serrano, G., Beach, T. G., & Doyle, K. P. (2016). Multiplex immunoassay characterization and species comparison of inflammation in acute and non-acute ischemic infarcts in human and mouse brain tissue. *Acta Neuropathologica Communications*, *4*(1), 100. https://doi.org/10.1186/s40478-016-0371-y

Noble, K. V., Liu, T., Matthews, L. J., Schulte, B. A., & Lang, H. (2019). Age-related changes in immune cells of the human cochlea. *Frontiers in Neurology*, *10*(AUG). https://doi.org/10.3389/fneur.2019.00895

Nwadiugwu, M. (2022). RNA-seq analysis of phagocytic cells from murine epididymal white adipose tissue shows immunosenescence and age-related phosphorus metabolism. *Human Cell*, *35*(2), 572–582. https://doi.org/10.1007/s13577-021-00663-4

O’Donnell, C., Migliore, E., Grandi, F. C., Koltsov, J., Lingampalli, N., Cisar, C., Indelli, P. F., Sebastiano, V., Robinson, W. H., Bhutani, N., & Chu, C. R. (2019). Platelet-Rich Plasma (PRP) From Older Males With Knee Osteoarthritis Depresses Chondrocyte Metabolism and Upregulates Inflammation. *Journal of Orthopaedic Research*, *37*(8), 1760–1770. https://doi.org/10.1002/jor.24322

Pascal, L. E., Igarashi, T., Mizoguchi, S., Chen, W., Rigatti, L. H., Madigan, C. G., Dhir, R., Bushman, W., DeFranco, D. B., Yoshimura, N., & Wang, Z. (2022). E-cadherin deficiency promotes prostate macrophage inflammation and bladder overactivity in aged male mice. *Aging*, *14*(7), 2945–2965. https://doi.org/10.18632/aging.203994

Patsalos, A., Simandi, Z., Hays, T. T., Peloquin, M., Hajian, M., Restrepo, I., Coen, P. M., Russell, A. J., & Nagy, L. (2018). In vivo GDF3 administration abrogates aging related muscle regeneration delay following acute sterile injury. *Aging Cell*, *17*(5). https://doi.org/10.1111/acel.12815

Pattabiraman, G., Palasiewicz, K., & Ucker, D. S. (2016). Toll-like Receptor function of murine macrophages, probed by cytokine induction, is biphasic and is not impaired globally with age. *Mechanisms of Ageing and Development*, *157*, 44–59. https://doi.org/10.1016/j.mad.2016.07.008

Pinto, A., Godwin, J., Chandran, A., Hersey, L., Ilinykh, A., Debuque, R., Wang, L., & Rosenthal, N. (2014). Age-related changes in tissue macrophages precede cardiac functional impairment. *Aging*, *6*(5), 399–413.

Porrini, V., Mota, M., Parrella, E., Bellucci, A., Benarese, M., Faggi, L., Tonin, P., Spano, P. F., & Pizzi, M. (2017). Mild inflammatory profile without gliosis in the c-rel deficient mouse modeling a late-onset parkinsonism. *Frontiers in Aging Neuroscience*, *9*(JUL). https://doi.org/10.3389/fnagi.2017.00229

Przybyla, B., Gurley, C., Harvey, J. F., Bearden, E., Kortebein, P., Evans, W. J., Sullivan, D. H., Peterson, C. A., & Dennis, R. A. (2006). Aging alters macrophage properties in human skeletal muscle both at rest and in response to acute resistance exercise. *Experimental Gerontology*, *41*(3), 320–327. https://doi.org/10.1016/j.exger.2005.12.007

Reidy, P. T., Lindsay, C. C., McKenzie, A. I., Fry, C. S., Supiano, M. A., Marcus, R. L., LaStayo, P. C., & Drummond, M. J. (2018). Aging-related effects of bed rest followed by eccentric exercise rehabilitation on skeletal muscle macrophages and insulin sensitivity. *Experimental Gerontology*, *107*, 37–49. https://doi.org/10.1016/j.exger.2017.07.001

Reidy, P. T., McKenzie, A. I., Mahmassani, Z. S., Petrocelli, J. J., Nelson, D. B., Lindsay, C. C., Gardner, J. E., Morrow, V. R., Keefe, A. C., Huffaker, T. B., Stoddard, G. J., Kardon, G., O’Connell, R. M., & Drummond, M. J. (2019). Aging impairs mouse skeletal muscle macrophage polarization and musclespecific abundance during recovery from disuse. *American Journal of Physiology - Endocrinology and Metabolism*, *317*(1), E85–E98. https://doi.org/10.1152/ajpendo.00422.2018

Renshaw, M., Rockwell, J., Engleman, C., Gewirtz, A., Katz, J., & Sambhara, S. (2002). Cutting Edge: Impaired Toll-Like Receptor Expression and Function in Aging. *The Journal of Immunology*, *169*(9), 4697–4701. https://doi.org/10.4049/jimmunol.169.9.4697

Rojas, J. X. S., Frutos, M. G. S., Horrillo, D., Lauzurica, N., Oliveros, E., Carrascosa, J. M., Fernández-Agulló, T., & Ros, M. (2016). Differential Development of Inflammation and Insulin Resistance in Different Adipose Tissue Depots Along Aging in Wistar Rats: Effects of Caloric Restriction. *Journals of Gerontology - Series A Biological Sciences and Medical Sciences*, *71*(3), 310–322. https://doi.org/10.1093/gerona/glv117

Rollo, E. E., & Denhardt, D. T. (1996). Differential effects of osteopontin on the cytotoxicity activity of macrophages from young and old mice. *Immunology* , *88*, 642–647.

Rosa, L. F. B. P. C., De Almeida, A. F., Safi, D. A., Curi, R., & Rosa, C. (1993). Metabolic and Functional Changes in Lymphocytes and Macrophages as Induced by Ageing. In *Physiology & Behavior* (Vol. 53).

Runyan, C. E., Welch, L. C., Lecuona, E., Shigemura, M., Amarelle, L., Abdala-Valencia, H., Joshi, N., Lu, Z., Nam, K., Markov, N. S., McQuattie-Pimentel, A. C., Piseaux-Aillon, R., Politanska, Y., Sichizya, L., Watanabe, S., Williams, K. J. N., Budinger, G. R. S., Sznajder, J. I., & Misharin, A. V. (2020). Impaired phagocytic function in CX3CR1+ tissue-resident skeletal muscle macrophages prevents muscle recovery after influenza A virus-induced pneumonia in old mice. *Aging Cell*, *19*(9). https://doi.org/10.1111/acel.13180

Saez, M. C., Garcia, J. J., De La Fuente, M., & Ortega, E. (2002). Modulation of superoxide anion levels of macrophages from young-adult and old mice by the norepinephrine metabolite, 4-hydroxy-3-methoxyphenyl-glycol. *Experimental Gerontology*, *37*, 395–400. www.elsevier.com/locate/expgero

Santeford, A. C., Lee, A. Y., Sene, A., Hassman, L. M., Sergushichev, A. A., Loginicheva, E., Artyomov, M. N., Ruzycki, P. A., & Apte, R. S. (2021). Loss of mir146b with aging contributes to inflammation and mitochondrial dysfunction in thioglycollate-elicited peritoneal macrophages. *ELife*, *10*. https://doi.org/10.7554/eLife.66703

Sato, S., Sakurai, T., Ogasawara, J., Takahashi, M., Izawa, T., Imaizumi, K., Taniguchi, N., Ohno, H., & Kizaki, T. (2014). A Circadian Clock Gene, Rev-erbα, Modulates the Inflammatory Function of Macrophages through the Negative Regulation of Ccl2 Expression . *The Journal of Immunology*, *192*(1), 407–417. https://doi.org/10.4049/jimmunol.1301982

Schneider, C. P., Schwacha, M. G., & Chaudry, I. H. (2007). Impact of sex and age on bone marrow immune responses in a murine model of trauma-hemorrhage. *J Appl Physiol*, *102*, 113–121. https://doi.org/10.1152/japplphysiol.00848.2006.-Although

Sebastián, C., Herrero, C., Serra, M., Lloberas, J., Blasco, M. A., & Celada, A. (2009). Telomere Shortening and Oxidative Stress in Aged Macrophages Results in Impaired STAT5a Phosphorylation. *The Journal of Immunology*, *183*(4), 2356–2364. https://doi.org/10.4049/jimmunol.0901131

Seicol, B. J., Lin, S., & Xie, R. (2022). Age-Related Hearing Loss Is Accompanied by Chronic Inflammation in the Cochlea and the Cochlear Nucleus. *Frontiers in Aging Neuroscience*, *14*, 846804. https://doi.org/10.3389/fnagi.2022.846804

Sene, A., Khan, A. A., Cox, D., Nakamura, R. E. I., Santeford, A., Kim, B. M., Sidhu, R., Onken, M. D., Harbour, J. W., Hagbi-Levi, S., Chowers, I., Edwards, P. A., Baldan, A., Parks, J. S., Ory, D. S., & Apte, R. S. (2013). Impaired cholesterol efflux in senescent macrophages promotes age-related macular degeneration. *Cell Metabolism*, *17*(4), 549–561. https://doi.org/10.1016/j.cmet.2013.03.009

Seo, J. Y., Kim, E. K., Lee, S. H., Park, K. C., Kim, K. H., Eun, H. C., & Chung, J. H. (2003). Enhanced expression of cylooxygenase-2 by UV in aged human skin in vivo. *Mechanisms of Ageing and Development*, *124*(8–9), 903–910. https://doi.org/10.1016/S0047-6374(03)00150-7

Shaik-Dasthagirisaheb, Y. B., Huang, N., Weinberg, E. O., Shen, S. S., Genco, C. A., & Gibson, F. C. (2015). Aging and contribution of MyD88 and TRIF to expression of TLR pathway-associated genes following stimulation with Porphyromonas gingivalis. *Journal of Periodontal Research*, *50*(1), 89–102. https://doi.org/10.1111/jre.12185

Shaik-Dasthagirisaheb, Yazdani B., Kantarci, A., & Gibson, F. C. (2010). Immune response of macrophages from young and aged mice to the oral pathogenic bacterium Porphyromonas gingivalis. *Immunity and Ageing*, *7*. https://doi.org/10.1186/1742-4933-7-15

Sheng, J., Yang, Y., Cui, Y., He, S., Wang, L., Liu, L., He, Q., Lv, T., Han, W., Yu, W., Hu, S., & Jin, J. (2018). M2 macrophage-mediated interleukin-4 signalling induces myofibroblast phenotype during the progression of benign prostatic hyperplasia article. *Cell Death and Disease*, *9*(755). https://doi.org/10.1038/s41419-018-0744-1

Sierra, A., Gottfried-Blackmore, A. C., Mcewen, B. S., & Bulloch, K. (2007). Microglia derived from aging mice exhibit an altered inflammatory profile. *GLIA*, *55*(4), 412–424. https://doi.org/10.1002/glia.20468

Sloboda, D. D., Brown, L. A., & Brooks, S. V. (2018). Myeloid cell responses to contraction-induced injury differ in muscles of young and old mice. *Journals of Gerontology - Series A Biological Sciences and Medical Sciences*, *73*(12), 1581–1590. https://doi.org/10.1093/gerona/gly086

Smallwood, H. S., López-Ferrer, D., & Squier, T. C. (2011). Aging enhances the production of reactive oxygen species and bactericidal activity in peritoneal macrophages by upregulating classical activation pathways. *Biochemistry*, *50*(45), 9911–9922. https://doi.org/10.1021/bi2011866

Son, M., Chung, W. J., Oh, S., Ahn, H., Choi, C. H., Hong, S., Park, K. Y., Son, K. H., & Byun, K. (2017). Age dependent accumulation patterns of advanced glycation end product receptor (RAGE) ligands and binding intensities between RAGE and its ligands differ in the liver, kidney, and skeletal muscle. *Immunity and Ageing*, *14*(1). https://doi.org/10.1186/s12979-017-0095-2

Spencer, N. F. L., & Daynes, R. A. (1997). IL-12 directly stimulates expression of IL-10 by CD5+ B cells and IL-6 by both CD5+ and CD5- B cells: possible involvement in age-associated cytokine dysregulation. *International Immunology*, *9*(5), 745–754.

Stahl, E. C., Delgado, E. R., Alencastro, F., LoPresti, S. T., Wilkinson, P. D., Roy, N., Haschak, M. J., Skillen, C. D., Monga, S. P., Duncan, A. W., & Brown, B. N. (2020). Inflammation and Ectopic Fat Deposition in the Aging Murine Liver Is Influenced by CCR2. *American Journal of Pathology*, *190*(2), 372–387. https://doi.org/10.1016/j.ajpath.2019.10.016

Stanojević, S., Kovačević-Jovanović, V., Dimitrijević, M., Vujić, V., Ćuruvija, I., Blagojević, V., & Leposavić, G. (2015). Unopposed Estrogen Supplementation/Progesterone Deficiency in Post-Reproductive Age Affects the Secretory Profile of Resident Macrophages in a Tissue-Specific Manner in the Rat. *American Journal of Reproductive Immunology*, *74*(5), 445–456. https://doi.org/10.1111/aji.12424

Stewart, A. N., Lowe, J. L., Glaser, E. P., Mott, C. A., Shahidehpour, R. K., McFarlane, K. E., Bailey, W. M., Zhang, B., & Gensel, J. C. (2021). Acute inflammatory profiles differ with sex and age after spinal cord injury. *Journal of Neuroinflammation*, *18*(1). https://doi.org/10.1186/s12974-021-02161-8

Stout-Delgado, H. W., Cho, S. J., Chu, S. G., Mitzel, D. N., Villalba, J., El-Chemaly, S., Ryter, S. W., Choi, A. M. K., & Rosas, I. O. (2016). Age-dependent susceptibility to pulmonary fibrosis is associated with NLRP3 inflammasome activation. *American Journal of Respiratory Cell and Molecular Biology*, *55*(2), 252–263. https://doi.org/10.1165/rcmb.2015-0222OC

Stout, R. D., Jiang, C., Matta, B., Tietzel, I., Watkins, S. K., & Suttles, J. (2005). Macrophages Sequentially Change Their Functional Phenotype in Response to Changes in Microenvironmental Influences. *The Journal of Immunology*, *175*(1), 342–349. https://doi.org/10.4049/jimmunol.175.1.342

Stratton, J. A., Eaton, S., Rosin, N. L., Jawad, S., Holmes, A., Yoon, G., Midha, R., & Biernaskie, J. (2020). Macrophages and Associated Ligands in the Aged Injured Nerve: A Defective Dynamic That Contributes to Reduced Axonal Regrowth. *Frontiers in Aging Neuroscience*, *12*. https://doi.org/10.3389/fnagi.2020.00174

Suzuki, M., Betsuyaku, T., Ito, Y., Nagai, K., Nasuhara, Y., Kaga, K., Kondo, S., & Nishimura, M. (2008). Down-regulated NF-E2-related factor 2 in pulmonary macrophages of aged smokers and patients with chronic obstructive pulmonary disease. *American Journal of Respiratory Cell and Molecular Biology*, *39*(6), 673–682. https://doi.org/10.1165/rcmb.2007-0424OC

Takahashi, R., Ishigami, A., Kobayashi, Y., & Nagata, K. (2016). Skewing of peritoneal resident macrophages toward M1-like is involved in enhancement of inflammatory responses induced by secondary necrotic neutrophils in aged mice. *Cellular Immunology*, *304*–*305*, 44–48. https://doi.org/10.1016/j.cellimm.2016.03.001

Takahashi, R., Totsuka, S., Ishigami, A., Kobayashi, Y., & Nagata, K. (2016). Attenuated phagocytosis of secondary necrotic neutrophils by macrophages in aged and SMP30 knockout mice. *Geriatrics and Gerontology International*, *16*(1), 135–142. https://doi.org/10.1111/ggi.12436

Tang, Y., Di Pietro, L., Feng, Y., & Wang, X. (2000). Increased TNF-a and PGI2 , but not NO release from macrophages in 18-month-old rats. *Mechanisms of Ageing and Development*, *114*, 79–88. www.elsevier.com/locate/mechagedev

Terao, A., Apte-Deshpande, A., Dousman, L., Morairty, S., Eynon, B. P., Kilduff, T. S., & Freund, Y. R. (2002). Immune response gene expression increases in the aging murine hippocampus. *Journal of Neuroimmunology*, *132*, 99–112. www.elsevier.com/locate/jneuroim

Thevaranjan, N., Puchta, A., Schulz, C., Naidoo, A., Szamosi, J. C., Verschoor, C. P., Loukov, D., Schenck, L. P., Jury, J., Foley, K. P., Schertzer, J. D., Larché, M. J., Davidson, D. J., Verdú, E. F., Surette, M. G., & Bowdish, D. M. E. (2017). Age-Associated Microbial Dysbiosis Promotes Intestinal Permeability, Systemic Inflammation, and Macrophage Dysfunction. *Cell Host and Microbe*, *21*(4), 455-466.e4. https://doi.org/10.1016/j.chom.2017.03.002

Throsby, M., Yang, Z., Lee, D., Huang, W., Copolov, D. L., & Lim, A. T. (1993). Coexpression of Atrial Natriuretic Factor and Beta-Endorphin in a Subpopulation of Rat Splenic Macrophages: Age-Related Differences. *Endocrinology*, *133*(6), 2889–2896. https://academic.oup.com/endo/article/133/6/2889/3036077

Tobin, S. W., Alibhai, F. J., Wlodarek, L., Yeganeh, A., Millar, S., Wu, J., Li, S. hong, Weisel, R. D., & Li, R. K. (2021). Delineating the relationship between immune system aging and myogenesis in muscle repair. *Aging Cell*, *20*(2). https://doi.org/10.1111/acel.13312

Trott, D. W., Henson, G. D., Ho, M. H. T., Allison, S. A., Lesniewski, L. A., & Donato, A. J. (2018). Age-related arterial immune cell infiltration in mice is attenuated by caloric restriction or voluntary exercise. *Experimental Gerontology*, *109*, 99–107. https://doi.org/10.1016/j.exger.2016.12.016

Uchida, M., Horii, N., Hasegawa, N., Fujie, S., Oyanagi, E., Yano, H., & Iemitsu, M. (2019). Gene Expression Profiles for Macrophage in Tissues in Response to Different Exercise Training Protocols in Senescence Mice. *Frontiers in Sports and Active Living*, *1*. https://doi.org/10.3389/fspor.2019.00050

Umehara, T., Winstanley, Y. E., Andreas, E., Morimoto, A., Williams, E. J., Smith, K. M., Carroll, J., Febbraio, M. A., Shimada, M., Russell, D. L., & Robker, R. L. (2022). Female reproductive life span is extended by targeted removal of fibrotic collagen from the mouse ovary. *Science Advances*, *8*(24). https://doi.org/10.1126/sciadv.abn4564

Verschoor, C. P., Johnstone, J., Loeb, M., Bramson, J. L., & Bowdish, D. M. E. (2014). Anti-pneumococcal deficits of monocyte-derived macrophages from the advanced-age, frail elderly and related impairments in PI3K-AKT signaling. *Human Immunology*, *75*(12), 1192–1196. https://doi.org/10.1016/j.humimm.2014.10.004

Vi, L., Baht, G. S., Soderblom, E. J., Whetstone, H., Wei, Q., Furman, B., Puviindran, V., Nadesan, P., Foster, M., Poon, R., White, J. P., Yahara, Y., Ng, A., Barrientos, T., Grynpas, M., Mosely, M. A., & Alman, B. A. (2018). Macrophage cells secrete factors including LRP1 that orchestrate the rejuvenation of bone repair in mice. *Nature Communications*, *9*(1). https://doi.org/10.1038/s41467-018-07666-0

Vida, C., de Toda, I. M., Cruces, J., Garrido, A., Gonzalez-Sanchez, M., & De la Fuente, M. (2017). Role of macrophages in age-related oxidative stress and lipofuscin accumulation in mice. *Redox Biology*, *12*, 423–437. https://doi.org/10.1016/j.redox.2017.03.005

Vineeta, K., Sodhi, A., & Singh, S. M. (1999). Age-Dependent Alterations in the Tumoricidal Functions of Tumor-Associated Macrophages. *Tumor Biology*, *20*, 30–43. http://biomednet.com/karger

Vivian, G. K., da Silva, R. O., Santos, A. C. A., Hastreiter, A. A., Dias, C. C., Makiyama, E. N., Borelli, P., de Oliveira Rodrigues, C., & Fock, R. A. (2023). The interaction between aging and protein malnutrition modulates peritoneal macrophage function: An experimental study in male mice. *Experimental Gerontology*, *171*, 112025. https://doi.org/10.1016/j.exger.2022.112025

Vtetvicka, V., Tlaskalova-Hogenova, H., Fornusek, L., Rihova, B., & Holant, V. (1987). Membrane and functional characterization of lymphoid and macrophage populations of Peyer’s patches from adult and aged mice. In *Immunology* (Vol. 62).

Vttvicka, V., Forniojsek, L., & Zidkova, J. (1985). The expression of Fc and complement receptors in young, adult and aged mice. In *Immunology* (Vol. 56).

Vu, R., Jin, S., Sun, P., Haensel, D., Nguyen, Q. H., Dragan, M., Kessenbrock, K., Nie, Q., & Dai, X. (2022). Wound healing in aged skin exhibits systems-level alterations in cellular composition and cell-cell communication. *Cell Reports*, *40*(5), 111155. https://doi.org/10.1016/j.celrep.2022.111155

Walker, A. E., Breevoort, S. R., Durrant, J. R., Liu, Y., Machin, D. R., Dobson, P. S., Nielson, E. I., Meza, A. J., Islam, M. T., Donato, A. J., & Lesniewski, L. A. (2019). The pro-atherogenic response to disturbed blood flow is increased by a western diet, but not by old age. *Scientific Reports*, *9*(1). https://doi.org/10.1038/s41598-019-39466-x

Wallace, P. K., Eisensteinb, T. K., Meissler, J. J. B., & Morahana, P. S. (1995). Decreases in macrophage mediated antitumor activity with aging. *Mechanisms of Ageing and Development*, *77*, 169–184.

Wang, C. Q., Udupa, K. B., Xiao, H., & Lipschitz, D. A. (1995). Effect of age on marrow macrophage number and function. *Aging (Milan, Italy)*, *7*(5), 379–384. https://doi.org/10.1007/BF03324349

Wang, Ying, Wehling-Henricks, M., Welc, S. S., Fisher, A. L., Zuo, Q., & Tidball, J. G. (2019). Aging of the immune system causes reductions in muscle stem cell populations, promotes their shift to a fibrogenic phenotype, and modulates sarcopenia. *FASEB Journal*, *33*(1), 1415–1427. https://doi.org/10.1096/fj.201800973R

Wang, Yuan, Zhu, S., Wei, W., Tu, Y., Chen, C., Song, J., Li, J., Wang, C., Xu, Z., & Sun, S. (2020). Interleukin-6 knockout reverses macrophage differentiation imbalance and alleviates cardiac dysfunction in aging mice. *Aging*, *12*(20), 20184–20197.

Werneck-Gomes, H., Campolina-Silva, G. H., Maria, B. T., Barata, M. C., Mahecha, G. A. B., Hess, R. A., & Oliveira, C. A. (2020). Tumor-Associated Macrophages (TAM) are recruited to the aging prostate epithelial lesions and become intermingled with basal cells. *Andrology*, *8*(5), 1375–1386. https://doi.org/10.1111/andr.12783

Wolfe, H., Minogue, A. M., Rooney, S., & Lynch, M. A. (2018). Infiltrating macrophages contribute to age-related neuroinflammation in C57/BL6 mice. *Mechanisms of Ageing and Development*, *173*, 84–91. https://doi.org/10.1016/j.mad.2018.05.003

Wong, C. K., Smith, C. A., Sakamoto, K., Kaminski, N., Koff, J. L., & Goldstein, D. R. (2017). Aging Impairs Alveolar Macrophage Phagocytosis and Increases Influenza-Induced Mortality in Mice. *The Journal of Immunology*, *199*(3), 1060–1068. https://doi.org/10.4049/jimmunol.1700397

Wu, D., Ren, Z., Pae, M., Han, S. N., & Meydani, S. N. (2013). Diet-induced obesity has a differential effect on adipose tissue and macrophage inflammatory responses of young and old mice. *BioFactors*, *39*(3), 326–333. https://doi.org/10.1002/biof.1075

Wung Chung, K., Oh Jeong, H., Lee, B., Park, D., Hyun Kim, D., Ja Choi, Y., Kyeong Lee, E., Mok Kim, K., Whoun Park, J., Pal Yu, B., & Young Chung, H. (2017). Involvement of NF-κBIZ and related cytokines in age-associated renal fibrosis. *Oncotarget*, *8*(5), 7315–7327. www.impactjournals.com/oncotarget/

Yamada, C., Beron-Pelusso, C., Algazzaz, N., Heidari, A., Luz, D., Rawas-Qalaji, M., Toderas, I., Mascarenhas, A. K., Kawai, T., & Movila, A. (2019). Age-dependent effect between MARCO and TLR4 on PMMA particle phagocytosis by macrophages. *Journal of Cellular and Molecular Medicine*, *23*(8), 5827–5831. https://doi.org/10.1111/jcmm.14494

Yang, Y., Ma, Y., Han, W., Li, J., Xiang, Y., Liu, F., Ma, X., Zhang, J., Fu, Z., Su, Y.-D., Du, X.-J., Gao, X.-M., & Gao, X. (2008). Age-related differences in postinfarct left ventricular rupture and remodeling. *Am J Physiol Heart Circ Physiol*, *294*, 1815–1822. https://doi.org/10.1152/ajpheart.00831.2007.-Cardiac

Yokozeki, Y., Kawakubo, A., Miyagi, M., Kuroda, A., Sekiguchi, H., Inoue, G., Takaso, M., & Uchida, K. (2021). Reduced TGF- β Expression and CD206-Positive Resident Macrophages in the Intervertebral Discs of Aged Mice. *BioMed Research International*, *2021*. https://doi.org/10.1155/2021/7988320

Yoon, P., Keylock, K. T., Hartman, M. E., Freund, G. G., & Woods, J. A. (2004). Macrophage hypo-responsiveness to interferon-γ in aged mice is associated with impaired signaling through Jak-STAT. *Mechanisms of Ageing and Development*, *125*(2), 137–143. https://doi.org/10.1016/j.mad.2003.11.010

Zandi, S., Nakao, S., Chun, K. H., Fiorina, P., Sun, D., Arita, R., Zhao, M., Kim, E., Schueller, O., Campbell, S., Taher, M., Melhorn, M. I., Schering, A., Gatti, F., Tezza, S., Xie, F., Vergani, A., Yoshida, S., Ishikawa, K., … Hafezi-Moghadam, A. (2015). ROCK-Isoform-Specific Polarization of Macrophages Associated with Age-Related Macular Degeneration. *Cell Reports*, *10*(7), 1173–1186. https://doi.org/10.1016/j.celrep.2015.01.050

Zhang, B., Bailey, W. M., McVicar, A. L., & Gensel, J. C. (2016). Age increases reactive oxygen species production in macrophages and potentiates oxidative damage after spinal cord injury. *Neurobiology of Aging*, *47*, 157–167. https://doi.org/10.1016/j.neurobiolaging.2016.07.029

Zhang, B., Bailey, W. M., McVicar, A. L., Stewart, A. N., Veldhorst, A. K., & Gensel, J. C. (2019). Reducing age-dependent monocyte-derived macrophage activation contributes to the therapeutic efficacy of NADPH oxidase inhibition in spinal cord injury. *Brain, Behavior, and Immunity*, *76*, 139–150. https://doi.org/10.1016/j.bbi.2018.11.013

Zhang, C., Cheng, N., Qiao, B., Zhang, F., Wu, J., Liu, C., Li, Y., & Du, J. (2020). Age-related decline of interferon-gamma responses in macrophage impairs satellite cell proliferation and regeneration. *Journal of Cachexia, Sarcopenia and Muscle*, *11*(5), 1291–1305. https://doi.org/10.1002/jcsm.12584

Zhang, Q., Kusaka, Y., & Donaldson, K. (2000). Comparative injurious and proinflammatory effects of three ultrafine metals in macrophages from young and old rats. *Inhalation Toxicology*, *12*(SUPPL. 3), 267–273. https://doi.org/10.1080/08958378.2000.11463222

Zhang, Y., Tang, L. hua, Lu, J., Xu, L. ming, Cheng, B. li, & Xiong, J. yu. (2021). ABT-263 enhanced bacterial phagocytosis of macrophages in aged mouse through Beclin-1-dependent autophagy. *BMC Geriatrics*, *21*(1). https://doi.org/10.1186/s12877-021-02173-2

Zhang, Z., Schlamp, F., Huang, L., Clark, H., & Brayboy, L. (2020). Inflammaging is associated with shifted macrophage ontogeny and polarization in the aging mouse ovary. *Reproduction*, *159*(3), 325–337. https://doi.org/10.1530/REP-19-0330

Zhao, C., Li, W. W., & Franklin, R. J. M. (2006). Differences in the early inflammatory responses to toxin-induced demyelination are associated with the age-related decline in CNS remyelination. *Neurobiology of Aging*, *27*(9), 1298–1307. https://doi.org/10.1016/j.neurobiolaging.2005.06.008

Zhao, H., Roychoudhury, J., Doggett, T. A., Apte, R. S., & Ferguson, T. A. (2013). Age-dependent changes in fasl (CD95L) modulate macrophage function in a model of age-related macular degeneration. *Investigative Ophthalmology and Visual Science*, *54*(8), 5321–5331. https://doi.org/10.1167/iovs.13-12122

Zhong, W., Rao, Z., Rao, J., Han, G., Wang, P., Jiang, T., Pan, X., Zhou, S., Zhou, H., & Wang, X. (2020). Aging aggravated liver ischemia and reperfusion injury by promoting STING-mediated NLRP3 activation in macrophages. *Aging Cell*, *19*(8). https://doi.org/10.1111/acel.13186
